# Leveraging the microbiome to understand clinical heterogeneity in depression: findings from the T-RAD study

**DOI:** 10.1038/s41398-023-02416-3

**Published:** 2023-04-28

**Authors:** Cherise R. Chin Fatt, Sarah Asbury, Manish K. Jha, Abu Minhajuddin, Sangita Sethuram, Taryn Mayes, Sidney H. Kennedy, Jane A. Foster, Madhukar H. Trivedi

**Affiliations:** 1 Center for Depression Research and Clinical Care, Peter O’Donnell Jr. Brain Institute and the Department of Psychiatry, University of Texas Southwestern Medical Center, Dallas, TX USA; 2 Department of Psychiatry & Behavioural Neurosciences, McMaster University, Hamilton, ON Canada; 3 Department of Psychiatry, University of Toronto and Centre for Depression and Suicide Studies, Unity Health, Toronto, ON Canada

**Keywords:** Predictive markers, Depression

## Abstract

Alterations in the gut microbiome have been linked to a variety of mental illnesses including anxiety and depression. This study utilized advanced bioinformatics tools that integrated both the compositional and community nature of gut microbiota to investigate how gut microbiota influence clinical symptoms in a sample of participants with depression. Gut microbiota of 179 participants with major depressive disorder (MDD) in the Texas Resilience Against Depression (T-RAD) study were analyzed by 16S rRNA gene sequencing of stool samples. Severity of anxiety, depression, and anhedonia symptoms were assessed with General Anxiety Disorder – 7 item scale, Patient Health 9-item Questionnaire, and Dimensional Anhedonia Rating Scale, respectively. Using weighted correlation network analysis, a data-driven approach, three co-occurrence networks of bacterial taxa were identified. One of these co-occurrence networks was significantly associated with clinical features including depression and anxiety. The hub taxa associated with this co-occurrence module –one *Ruminococcaceae* family taxon, one *Clostridiales vadinBB60 group* family taxon, and one *Christencenellaceae* family taxon– were connected to several additional butyrate-producing bacteria suggesting that deficits in butyrate production may contribute to clinical symptoms. Therefore, by considering the community nature of the gut microbiome in a real world clinical sample, this study identified a gut microbial co-occurrence network that was significantly associated with clinical anxiety in a cohort of depressed individuals.

## Introduction

Harnessing the potential of the microbiome for precision medicine approaches in psychiatry is at the forefront of clinical neuroscience. Much of the attention in this area has focused on the trillions of resident microbes in the gastrointestinal tract—referred to as the gut microbiome. The gut microbiome has been implicated in the etiology and pathogenesis of a number of diseases and disorders including: inflammatory bowel disease, irritable bowel syndrome, celiac disease, asthma, obesity, cancer, neurodegenerative diseases, autoimmune diseases, as well as mental health disorders, including, anxiety, schizophrenia, and depression [1–10]. Accumulating evidence has demonstrated compositional differences in gut microbiota between healthy individuals and those with mood and anxiety disorders. Across studies, several bacterial genera have been reported to be enriched in healthy individuals or enriched in major depressive disorder (MDD) [11–17], bipolar disorder (BP) [18–21], and general anxiety disorder (GAD) [22, 23]. Moreover, studies have identified associations between specific gut microbial taxa and clinical symptoms including anxiety [14, 22] and sleep quality [13], as well as the severity of disease [11, 12, 14, 15, 18, 19, 22]. This study examined the association between gut microbiota community composition and clinical symptoms, including anhedonia, depression, and anxiety, in individuals with a current or past diagnosis of depression enrolled in the Texas Resilience Against Depression (T-RAD) study [24].

To garner the potential of examining the microbiome in psychiatry, it is important to recognize that the gut microbiome is a diverse ecosystem in which microbial cross-feeding and competition for nutrient resources influence the stability, composition, and function of the gut microbial community. Most studies to date have considered associations between single bacterial taxa and clinical phenotype, which does not consider the dynamic and community nature of the gut microbiome. This overlooked feature may be a critical factor to understand how bacteria-host communication influences host physiology. To fill this gap, we took a novel approach using advanced bioinformatics tools that integrated both the compositional and community nature of gut microbiota. Specifically, weighted correlation network analysis (WCNA), a data-driven tool commonly used in genomic analyses, was employed to identify co-occurring networks of gut microbiota. WCNA examines the biological networks’ structure by defining clusters of highly correlated taxa, identifying highly connected hub taxa, and comparing the network topology generated by different adjacency matrices [25]. More importantly, WCNA is differentiated from other network methods by constructing co-occurrence networks using the topological overlap measure, which quantifies the extent to which taxa share common neighbors.

This report provides a data-driven analysis of 16S rRNA microbiome sequencing data to identify networks of co-occurring human gut microbial communities in a sample of patients with current or lifetime diagnosis of depression. Once identified, these networks were used to answer the following questions: (1) Are these co-occurring networks related to clinical symptoms (depression, anhedonia, anxiety)? (2) What are the hub taxa for the co-occurring networks and are these hub taxa clinically relevant? This work demonstrates a novel bioinformatic approach to generate a microbial signature using 16S rRNA sequencing data that can be applied in clinical psychiatric research.

## Methods

### Participants

Participants in this study were recruited as part of the Dallas 2 K (D2K) study, a component of Texas Resilience Against Depression Study (T-RAD) [24]. D2K follows participants who are 10 years of age and older and who have a current or past diagnosis of depression or bipolar disorder. Participants sign an institutional review board-approved informed consent form (University of Texas Southwestern Medical School Institutional Review Board) prior to initiation of any study-related procedure and sign an authorization for the use and disclosure of health information for research purposes (Health Insurance Portability and Accountability Act - HIPAA authorization). These studies are registered with clinicaltrials.gov (NCT02919280; NCT03458936). At study enrollment, participants receive comprehensive demographic and psychiatric assessment through a combination of self-report surveys and clinician-rated measures. Additional study details have been previously reported [24]. This analysis focused on adult participants (*n* = 179) with current or past diagnoses of major depressive disorder (MDD) who had clinical assessments and a stool sample at the same visit.

### Clinical assessments

The Patient Health Questionnaire (PHQ-9), a 9-item self-report questionnaire, used to assess depression severity (range 0 to 27 with higher scores between 20 and 27 reflecting severe depression) [26]. The General Anxiety Disorder —7 item scale (GAD-7) is a 7-item self-report questionnaire that measures anxiety symptoms [27]. The Dimensional Anhedonia Rating Scale (DARS), a 17-item self-report questionnaire, assesses anhedonia across four domains, namely, hobbies/past-times, food/drinks, social activities, and sensory experiences [28]. Total DARS score ranges from 0 to 68 with higher scores reflecting greater motivation, effort, and pleasure (i.e., less anhedonia).

### Fecal sample collection and storage

Study participants were given a stool sample kit at each in-person visit to collect the stool sample. Explicit instructions were given to the participants on how to collect the samples. Once collected (within seven days of an in-person visit), the biological specimens were either chilled or frozen (if the sample could be returned within 48 hours of collection) by the participant. Once returned to the Center for Depression Research and Clinical Care, the samples were frozen at −80 °C until sequencing. All stool samples were collected within 1 week of the participant’s clinical assessments.

#### 16S rRNA sequencing

Bacterial DNA was extracted from stool samples using methods previously described with some modifications [29]. 16S rRNA gene sequences were amplified according to published protocols with modifications outlined by Whelan and colleagues [30, 31], using PCR primers specific for the variable 3 (v3) and variable 4 (v4) regions of the 16 S ribosomal RNA (rRNA) encoding gene (341f–CCTACGGGNGGCWGCAG and 802r–GGACTACNVGGGTW TCTAAT′). 16S rRNA sequencing results were pre-processed using the DADA2 pipeline and amplicon sequence variants (ASVs) were annotated using the SILVA 128 reference database [32]. Samples were quality controlled for sequencing depth and α-diversity outliers. One sample was removed from the dataset for concurrent low sequencing depth (reads = 5466) and low α-diversity (Shannon Index = 2.00). Taxa abundance from the remaining 178 participants were used in downstream microbiome analyses.

#### 16 S rRNA analysis

##### Retain-resolve agglomeration

**Fig. 1:**
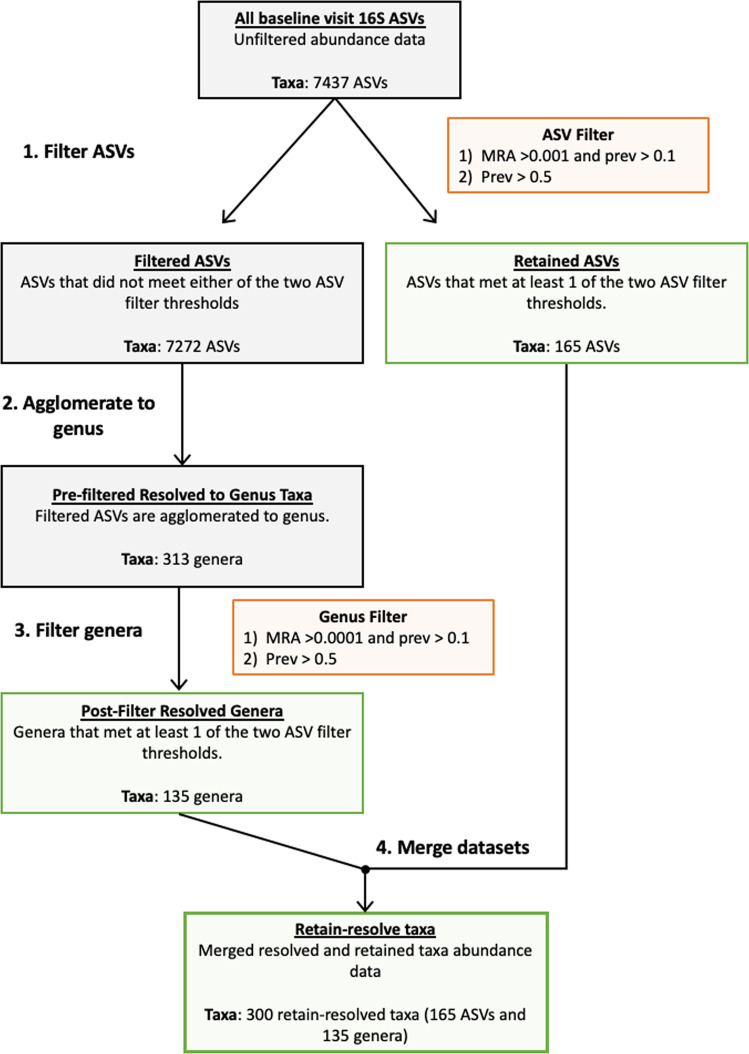
Retain-resolve agglomeration method. Mean relative abundance (MRA) and prevalence (prev) thresholds were set for filtering ASVs and genera. The final 16S taxa dataset contains a mix of ASV-level and genus-level taxa (Supplementary Table S1) that met MRA and prevalence thresholds, or prevalence thresholds alone.

Dimension reduction of quality controlled 16 S rRNA microbiome taxa was achieved using an in-house retain-resolve agglomeration strategy (Fig. 1). This strategy retains common amplicon sequence variants (ASVs) and resolves rare ASVs to filtered genus-level taxa. Initial number of unique ASVs was 7867. Criteria for ASV retention was set to prevalence >50% or mean relative abundance >0.1% and prevalence >10%. 165 ASVs met criteria and were retained in the analytical dataset. Remaining ASVs that did not meet the criteria were agglomerated to genus-level taxa using the *tax_glom* function in *phyloseq* in R [33]. Resolved genus taxa were then filtered by the criteria – prevalence >50% or mean relative abundance >0.01% and prevalence >10%. 135 agglomerated genus-level taxa met filtering criteria in the analytical dataset, for a total number of 300 retain-resolved taxa (Fig. 1, Supplementary Table S1). To preserve integrity of the center log ratio (CLR) transformation, we also generated an “Other taxa” label for each sample, which accounts for the proportion of taxa lost during genus-level filtering. All downstream analyses were performed using retain-resolved taxa.

##### Microbiome co-occurrence community

The microbiome co-occurrence network was generated from 300 retain-resolved taxa using weighted correlation network analysis (WCNA) using the WGCNA package in R [25]. Covariate-corrected networks were generated using CLR taxa abundances adjusted for age and BMI using empirical bayes-moderated linear regression *(empiricalBayesLM)* in the WGCNA package. A soft-threshold power (β) of 3 was selected using WGCNA guidelines to satisfy scale-free topology criteria. Minimum module size of 10, 15, or 25 were used to generate networks; each network was assessed for individual module stability. Merging similar modules was attempted, however merged module was equivalent to original module sets for each network. Module taxa abundance in each sample was summarized as the module eigentaxon, which is a single value calculated using the *moduleEigengenes* function in WCGNA. Participants with high abundance of a particular module will have a higher module eigentaxon value. Then, association between module eigentaxon and continuous clinical traits (age, PHQ-9 total score, GAD-7 total score) was determined using Pearson’s correlation coefficient according to WGCNA guidelines. Taxa in the 95th percentile for module membership were designated as hub taxa. Module membership was calculated by correlating taxa with each module eigentaxon. A higher module membership indicates a stronger relationship between the taxon and module abundance across samples. Module membership is also a proxy measure of taxa network node connectivity.

Taxa significance was defined here as the signed Pearson correlation between an individual taxa and clinical trait (GAD7, PHQ9, DARS). Correlation significance was adjusted using Benjamin-Hochberg correction. Higher taxa significance indicates that taxa is more biologically significant to the clinical trait. Moreover, high correlation between taxa significance and clinical trait suggests the module is robustly associated with the clinical trait. Lower correlation suggests a few select taxa within the module are likely driving the module’s association with the trait, rather than the microbial community. Correlation between module membership and absolute taxa significance – defined here as the unsigned correlation between individual taxa and clinical trait – is also provided for consistency with WCGNA’s methods.

##### WCNA module stability

WCNA module stability was assessed by applying similar principles used in clustering stability analysis previously described [34]. The module stability algorithm is summarized in Supplementary Fig. S1. First, the taxa belonging to the original WCNA network were determined. The dataset was then resampled with replacement 1000 times, and taxa assigned to each bootstrapped network module was determined. The Jaccard similarity of bootstrapped modules was compared to the original modules, and the bootstrapped module with highest Jaccard similarity value was matched to each original module. Module stability was defined as the mean Jaccard similarity of the 1000 bootstrapped modules assigned to the original module. Valid modules were those that had module stability greater than 0.5, akin to minimum definition of a cluster used in other cluster stability analyses [34].

##### Network visualization

The brown module network was visualized as a hard-threshold correlation network using Cytoscape (version 3.9.0) [35]. Network connections were generated using Pearson’s correlations coefficient between taxa calculated via the *rcorr* function in the Hmisc R package [36]. An arbitrary threshold of *r* > 0.35 was set to simplify Cytoscape visualization. Edges were sized according to the strength of the correlation between taxa, with the thinnest connections corresponding to correlation of *r* = 0.35. Taxa nodes were also sized and colored by their brown module membership. Finally, taxa in the Clostridiales order are represented by oval-shaped nodes, while taxa belonging to all other taxonomic orders are represented by hexagonal nodes. A simple network visualization was also generated with a weak correlation threshold of *r* > 0.2. Taxa that did not connect to the main network after hard thresholding were removed from the visualization.

##### ALDEx and Random Forest

ALDEx and random forest methods, which are often used in microbiome analysis, were employed to complement the results of the WCNA analytical approach and generate consensus taxa were associated with age, anxiety, and depression. Association between individual taxa and each continuous clinical trait (age, PHQ-9 total score, GAD-7 total score) was determined separately using generalized linear models via ALDEx2 *glm* function [37]. Taxa met criteria for ALDEx2 in our consensus analysis if they had an unadjusted p-value less than 0.05. Random forest regression was used to predict continuous clinical traits (age, PHQ-9 total score, GAD-7 total score) from 16S taxon CLR transformed abundances using the *randomForest* package in R [38]. Taxa met the criteria for random forest in our consensus analysis if they were ranked one of the top 20 important predictor variables for the clinical trait.

## Results

The analytical sample included 179 participants (124 female) with average age of 46.2 years (SD = 15.9). Briefly, 50% of participants had moderate or higher severity of depression (PHQ-9 total score *≥* 10), and 37% of participants had moderate or higher severity of anxiety (GAD-7 total score *≥* 10) at the time of sample collection and clinical assessment. See Table 1 for more detailed demographics and current mood state description of the analytical sample population.

**Table 1:** Demographic and current mood state characteristics of the participants.

Demographics		
	*n*	%
*Sex*		
Male	55	31.0
Female	124	69.0
*Race*		
White	133	74.3
Black/African American	26	14.5
Other	13	7.2
Unknown	7	3.9
*Ethnicity (Hispanic/ Non-Hispanic)*		
Yes	21	11.7
No	143	79.9
Unknown	15	8.4
	Mean	SD
Age in years^a^	46.2	15.9
Current Mood State		
	*n*	%
*PHQ-9*^*b*^		
None	45	25.7
Mild	42	23.5
Moderate	40	22.3
Severe	30	16.2
Very Severe	22	12.3
*GAD-7*^*b*^		
None	59	33.5
Mild	52	29.1
Moderate	39	21.8
Severe	29	15.6
DARS^b,c^	49.7	13.3
Non anhedonic	123	69.2
Anhedonic	48	26.3

^a^2 participants missing age in years.

^b^PHQ-9 cutoffs: None (0–4), Mild (5–9), Moderate (10–14), Severe (15–19), Very Severe (20 and above); GAD-7 cutoffs: None (0–4), Mild (5–9), Moderate (10–14), Severe (15 and above); DARS cutoffs: Non-Anhedonic (>44), Anhedonic (<= 44).

^c^8 participants missing DARS total score.

### Microbiome co-occurring communities

**Fig. 2:**
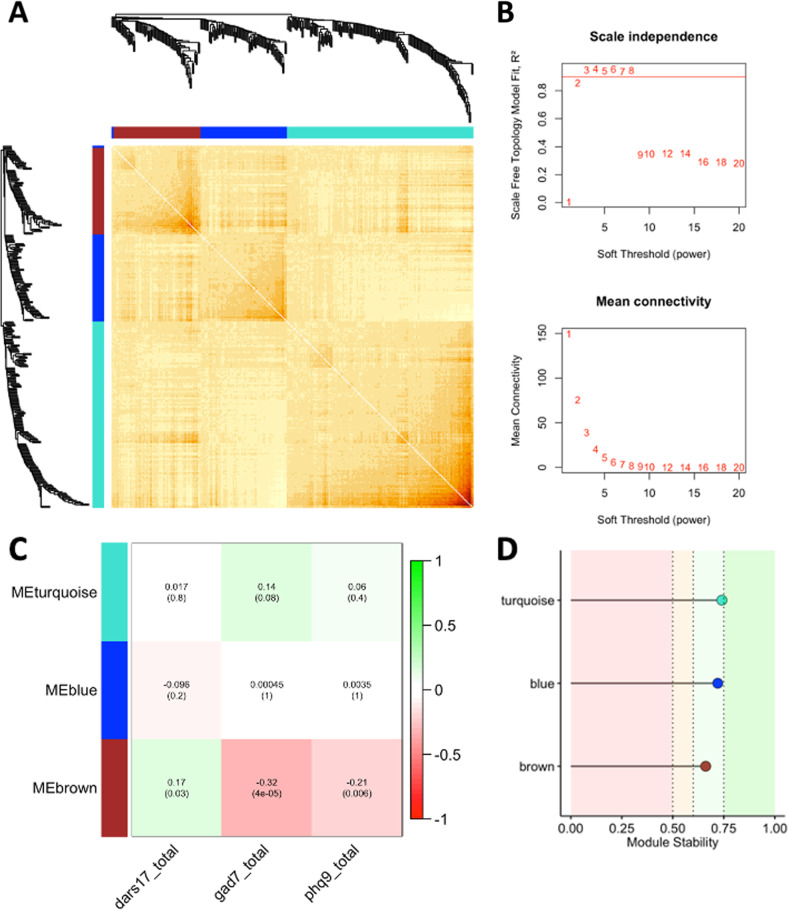
Weighted taxon correlation network analysis reveals a gut microbial network associated with anxiety, depression, and anhedonia clinical test scores after BMI and age correction. The network was generated using minimum module size of 25. **A** Weighted correlation network soft-threshold parameter selection criteria graphs. **B** Weighted taxon correlation network visualized using dendrogram and heatmap of Topological Overlap Matrix. Our clinical study has three robust human gut microbial modules. **C** Correlation of gut microbial networks with clinical mood status. Abundance of taxa in the brown network are negatively correlated with clinical measures of anxiety, depression, and anhedonia. **D** The human gut microbial communities form valid modules. Module stability was calculated using the mean Jaccard similarity from one thousand bootstrapped module taxa and the original module taxa.

WCNA identified three bacterial modules—labeled turquoise, blue and brown—which represent structured networks of co-occurring human gut microbial communities (Fig. 2). The three-module result was validated by comparing the module stability of the three modules and the module stability of two additional networks with varying minimum module size (Fig. 2, Supplementary Fig. S2). All three modules in the initial network formed stable modules, and were therefore selected for downstream analyses (Fig. 2). A significant assocation between module eigentaxon (ME) and clinical traits revealed that the brown module was significantly negatively correlated with anxiety, depression, and positively correlated with anhedonia (Fig. 2), such that lower abundance of this microbiota network (brown module) was associated with increased clinical symptoms. The microbial composition of the brown module was further investigated by identifying hub taxa and clinically significant taxa.

**Fig. 3:**
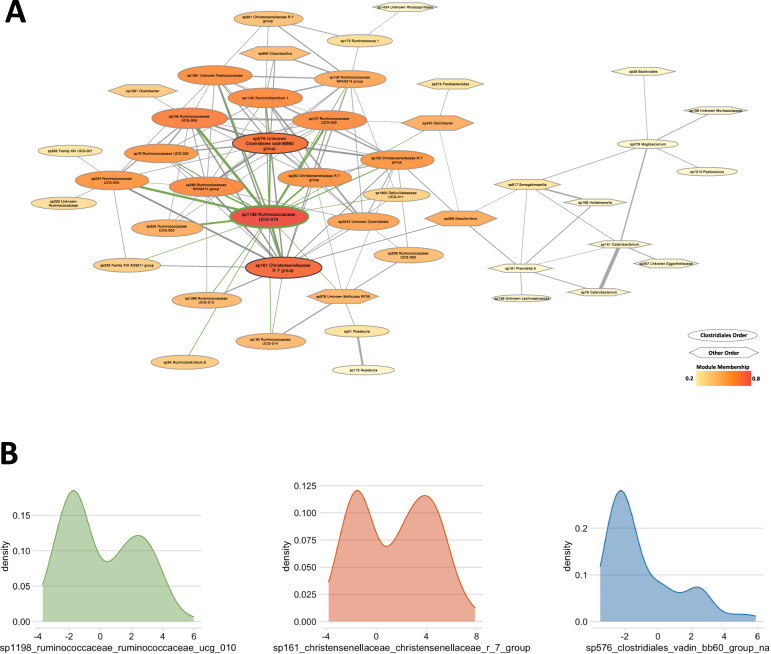
The brown human gut microbial network – which is positively associated with clinical scores of anxiety, depression, and anhedonia – is enriched and regulated by butyrate-producing human gut microbes. **A** Visualization of the brown gut microbial network as a hard-thresholded correlation network. Network connections were determined using Pearson correlation. Connections were pruned to taxa correlations where *R* > 0.35. Edges correspond to correlation strength, with the thinnest correlations corresponding to *r* = 0.35. Nodes are sized and colored by Brown module membership, and shapes are coded by taxonomic order. **B** Probability density function plots for hub taxa of the brown network. Hub taxa represent probable network regulators.

Module hub taxa were defined as the most highly connected taxa in the module’s network. The brown module hub taxa were genus-level taxa, including one *Ruminococcaceae* family taxon, one *Christencenellaceae* family taxon, and one *Clostridiales vadinBB60 group* family taxon (Fig. 3). Sp1198 *Ruminococcaceae UCG-010* and sp161 *Christensenellaceae R7 group* are both highly prevalent taxa in the population (Fig. 3, Supplementary Table S1). Sp1198 *Ruminococcaceae UCG-010* also has the highest brown module connectivity, as indicated by high module membership (Supplementary Table S2).

Brown module taxa that had Pearson correlations greater than 0.35 with at least one other brown module taxon were visualized in a hard-threshold correlation network (Fig. 3). For reference, the correlation network for the brown module that had correlations greater than 0.2 is provided in Supplemental Fig. S3. This visualization highlights the presence of several Ruminococcaceae family taxa in the brown module (see Supplementary Table S2 for full list of brown module taxa).

**Fig. 4:**
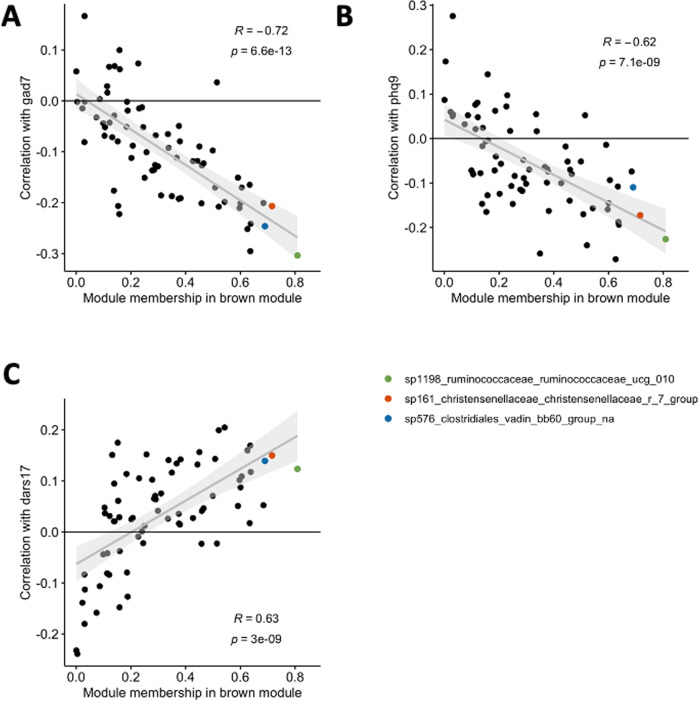
Association between signed brown module taxa significance and brown module membership. Taxa significance is defined here as the signed correlation between the taxa and clinical trait. Hub taxa for the brown module are highlighted as green, orange, or blue. **A** GAD7 taxa significance association with module membership. **B** PHQ9 taxa significance association with module membership. **C** DARS taxa significance association with module membership.

The association between taxa significance and module membership measures the robustness of the correlation between the clinical trait and bacterial community. Signed taxa significance for GAD-7 and taxa brown module membership had a strong negative correlation (*r* = −0.72, *p* = 6.6e−13; Fig. 4). The high correlation between taxa significance and module membership indicates increased confidence that the entire brown module bacterial community is associated with the clinical trait, rather than association being driven by individual non-hub taxa in the module [25]. Indeed, the hub taxa (visualized in color in Fig. 4) were among the most negatively correlated with GAD-7 score of all brown module taxa, indicating a durable and strong relationship between the brown module and anxiety. Hub taxa sp1198 *Ruminococcaceae UCG-010* and sp576 *Clostridiales vadinBB60 group unknown* were both significantly negatively associated with GAD-7 score (*p* = 0.005 and *p* = 0.027 respectively). Furthermore, brown module signed taxa significance is unidirectional, as all significant correlations between brown module taxa and GAD-7 score were negative (Fig. 4, Supplementary Table S2). This indicates that reduction of bacteria in the brown module community was uniformly associated with increased clinical anxiety scores.

Module membership and taxa significance were also associated for PHQ-9 and DARS, albeit to a lesser degree than GAD-7 (Fig. 4). However, signed taxa significance for PHQ-9 was not unidirectional with at least one positively correlated taxa, indicating a less robust relationship between clinical depression scores and the brown module (Fig. 4, Supplementary Table S2). DARS clinical scores were not significantly correlated with any brown taxa, indicating a weak relationship between the module and anhedonia. PHQ-9 was trending towards significantly negatively correlated (*p* = 0.053 and |r| = −0.23) with primary hub taxa sp1198 *Ruminococcaceae UCG-010* (Supplementary Table S2). Correlation between module membership and absolute taxa significance for all clinical traits—defined here as the unsigned correlation between taxon and clinical trait—is also provided for consistency with other WCNA pipelines and resulted in similar interpretations (Supplementary Fig. S4).

WCNA was also performed with CLR abundances without age and BMI correction. We identified similar associations between clinical symptoms with a module defined by primary hub taxa sp1198 *Ruminococcaceae UCG-010* (Supplementary Fig. S5, Supplementary Table S3). Overall, our WCNA results demonstrate three stable microbial co-occurrence modules, one of which that is significantly and robustly negatively associated with anxiety symptoms.

### ALDEx and Random Forest

Complementary analyses using ALDEx revealed several individual taxa that were significantly associated with continuous clinical traits (21 taxa with GAD-7, 19 taxa with PHQ-9, and 3 taxa with DARS) before correction (p value < 0.05; Supplementary Table S4). However, taxa associations did not survive multiple correction (Benjamini-Hochberg adjusted p_BH_ value < 0.05) (Supplementary Table S4, Supplementary Fig. S6). Additionally, random forest models trained to predict clinical trait values for GAD-7, PHQ-9, from patient CLR transformed abundances of individual taxa had low predictive accuracy on validation datasets (Supplementary Fig. S7). There was no predictive utility of microbial taxa for anhedonia clinical scores in our study (Supplementary Fig. S8).

To identify taxa of interest for potential biomarkers of depression, anxiety, or anhedonia we examined the consensus of all three analytical methods—WCNA, ALDEx2 and random forest. The taxa that met the criteria for significance in WCNA and either ALDEx2 or random forest methods are listed in consensus tables (Tables 2, 3). Brown module hub taxa sp1198 *Ruminococcaceae UCG-010* met all criteria for GAD-7 consensus taxa. Further, its association with GAD7 score remained significant after BH correction (Supplemental Table 2). Several additional taxa outside the brown module were also identified by meeting ALDEx2 and random forest criteria—the strongest individual taxa association being sp28 *Lachnospiraceae_NA* (β_GAD7_ = 0.28). Although these individual taxa did not survive multiple correction in our analyses, this list represents potential taxa that may be considered as potential microbiota biomarkers for anxiety symptoms. PHQ-9 did not have any brown module hub taxa that achieved consensus criteria. However, GAD7 and PHQ-9 shared several positively associated consensus taxa including positive associations with *Faecalitalea* at both genus (β_GAD7_ = 0.19, β_PHQ9_ = 0.13) and ASV-level (β_GAD7_ = 0.17, β_PHQ9_ = 0.15) and genus-level *Romboutsia* (β_GAD7_ = 0.17, β_PHQ9_ = 0.12). Genus-level *Christensenellacea* was also negatively associated with both GAD7 and PHQ9 (β_GAD7_ = -0.18, β_PHQ9_ = 0.15). No consensus taxa were identified for DARS.

**Table 2:** Consensus mood-associated taxa (GAD-7 score).

Taxa	Resolution	WCNA	Random Forest	Aldex	Aldex linear model		
					β	*p*	p.BH
**sp1198_Ruminococcaceae_UCG-010**	genus	YES	YES	YES	−0.22	<0.001	0.195
sp280_Ruminococcaceae_NK4A214_group	genus	YES	YES	YES	−0.22	0.004	0.503
sp1361_Peptococcaceae_NA	genus	YES	YES	YES	−0.14	0.027	0.777
sp380_Romboutsia	genus		YES	YES	0.17	0.015	0.695
sp524_Christensenellaceae_NA	genus		YES	YES	−0.18	0.001	0.243
sp372_Faecalitalea	genus		YES	YES	0.19	0.01	0.777
sp244_Bifidobacterium	genus		YES	YES	0.22	0.003	0.556
sp122_Lachnoclostridium	ASV		YES	YES	0.16	0.034	0.978
sp28_Lachnospiraceae_NA	ASV		YES	YES	0.28	0.001	0.354
sp109_Faecalitalea	ASV		YES	YES	0.17	0.035	0.963

**Table 3:** Consensus mood-associated taxa (PHQ-9 score).

Taxa	Resolution	WCNA	Random Forest	Aldex	Aldex Linear Model		
					β	p	p.BH
sp1361_Peptococcaceae_NA	genus	YES	YES	YES	−0.13	0.008	0.673
sp1802_Defluviitaleaceae_UCG-011	genus	YES	YES	YES	−0.12	0.019	0.769
sp524_Christensenellaceae_NA	genus		YES	YES	−0.15	0.002	0.294
sp129_Lachnoclostridium	ASV		YES	YES	0.15	0.013	0.864
sp299_Sutterella	genus		YES	YES	0.12	0.048	0.966
sp288_Megasphaera	genus		YES	YES	0.15	0.022	0.815
sp380_Romboutsia	genus		YES	YES	0.12	0.039	0.874
sp372_Faecalitalea	genus		YES	YES	0.13	0.04	0.973
sp109_Faecalitalea	ASV		YES	YES	0.15	0.026	0.907

## Discussion

In this sample of adults with depression, using 16S rRNA sequencing and clinical data, three co-occurrence communities of bacterial taxa were identified. One of these communities was significantly associated with clinical features including depression, anxiety, and anhedonia. Further, the microbial composition of this co-occurrence module was enriched with butyrate-producing bacteria. Importantly, these analyses demonstrate that applying weighted network correlation analysis to understand the community nature of the gut microbiome reveals stable microbiota networks in depressed individuals. Moreover, consideration of the bacterial community structure generated clinically relevant associations that were more robust than those generated by standard microbiome tools. Visualization of the clinically associated network demonstrated that numerous connections exist between co-occurring taxa and employing an integrative analytical approach may provide a more accurate and robust view of the community of bacterial associated with clinical symptoms.

While anxiety symptoms are co-morbid in both physical and mental illness, clinical research design does not adequately address the presence anxiety or other co-morbidities [39]. As such, translating clinical research findings to real world depressed individuals is challenging. The T-RAD study aims to better understand depression in a naturalist real world patient population [24]. The current study leveraged a data-driven microbiome approach to generate a more holistic and biologically specific classification of depressed participants. By doing so, we identify a clinically relevant co-occurring community of gut bacteria that was significantly associated with mood symptoms in a broad sample of individuals with current or previous depression. The clinically relevant co-occurrence module of bacteria was reduced in individuals with increased anxiety, depressive, or anhedonic symptoms and included several members of the *Ruminococcaceae* family. The primary hub taxa belonged to the *Ruminoccocaceae* family. In addition, several module members belong to the *Ruminococcaceae* family and were significantly associated with anxiety symptoms following multiple correction. Notably, reduced abundance of *Ruminococcaceae* taxa was associated with increased depressive symptoms in two large cohorts, the Rotterdam Study cohort & Amsterdam HELIUS cohort, with 1054 and 1539 depressed individuals, respectively [40] *Ruminococcaceae* family taxa are butyrate-producing bacteria in the human gut [41, 42]. Butyrate is a short-chain fatty acid that is a product of microbial fermentation. Reduced abundance of butyrate-producing bacteria have previously been reported in depressed individuals [11, 43]. Butyrate contributes to the maintenance of a healthy intestinal barrier and homeostatic immune responses [44]. Indeed, butyrate is a primary fuel source for intestinal epithelial cells supporting homeostatic proliferation of intestinal epithelial cells [44]. Intestinal epithelial cell-cell adhesion by tight junction proteins forms the intestinal barrier, which prevents translocation of bacteria and gut metabolites that induce deleterious host inflammation [44]. Butyrate helps maintain intestinal barrier integrity and biomarkers of intestinal permeability, including zonulin and intestinal fatty acid binding protein (IFAP), have been associated with depression and anxiety disorders [45]. Furthermore, butyrate promotes intestinal regulatory T cell differentiation and production of anti-inflammatory cytokines IL-10 and TGF-β, while concurrently decreasing mature antigen-presenting dendritic cells and levels of pro-inflammatory cytokines, demonstrating its direct importance in immune regulation [44]. A dysregulated immune response has been associated with greater depressive symptom severity [46, 47], resistance to commonly used antidepressants [48], and a higher likelihood of hospitalizations in depressed patients [49, 50]. Overall, the association of a reduced community of butyrate-producing bacterina with mood symptoms lends microbiota-immune regulation as an important target in investigating the causal link between gut microbiome, immune dysfunction, and depression as well as anxiety.

The second hub taxa of the clinically relevant microbiota module was a member of the recently identified *Christencenellaceae* family. The *Christencenellaceae* family of bacteria have been reported to be heritable as revealed by microbiome studies in twins, and have been linked to metabolic health and aging [51]. *Christencenellaceae* abundance has been associated with body mass index (BMI), with reduced abundance of *Christencenellaceae* in obese individuals (BMI > 30) compared to individuals with a normal BMI [51]. Reduced abundance of *Christencenellaceae* has also been reported in individuals with metabolic syndrome [52, 53]. Increased inflammation is associated with increased BMI, poor metabolic health, and depression.

The functions of hub taxa *Clostridiales Vadin BB60* family emerging. Recent studies indicate it may be a marker of gut-brain axis health. Depressed patients were reported to have lower *Clostridiales Vadin BB60* family abundance compared to healthy controls [11]. Additionally, abundance of *Clostridiales Vadin BB60* was a robust biomarker for microbiome-based Alzheimer’s treatment in mice [54]. The biological activity of *Clostridiales Vadin BB60* family in the gut lumen is unknown, however, these taxa have been linked to increased dopamine metabolites or serotonin precursors in the brainstem [55] and potentially contribute to microbiome-induced protection against experimentally induced colitis [56]. *Clostridiales Vadin BB60* family taxa belong to the *Clostridiales* order, and other *Clostridiales* species are associated with reduced gut lumen inflammation via T_reg_ expansion [57]. While not directly tested in the current study, it is plausible that the community of microbiota identified here in the brown co-occurrence module may represent a microbial community in healthy individuals that protects against inflammatory processes and at the same time prevents inflammation-related anxiety and depressive symptoms.

The significant relationship between anxiety and a gut microbial community identified in these analyses translates earlier neuroscience studies linking microbiota to anxiety-related behavior [58–62] to clinical anxiety. These landmark gut-brain axis studies demonstrated that microbiota influenced stress-reactivity and stress-related (e.g., anxiety-like) behaviors using germ-free mice and were a catalyst for neuroscientists to consider how microbes may influence brain function [60–63]. Since then, microbiome research in animal models has demonstrated a role for microbiota-brain communication in brain development, behavior, and brain function [64]. In the past 5 years, several reports have extended these preclinical findings to mood and anxiety disorders [11–23]. A recent large population study reported an association between the microbiome and quality of life and depression [43]. The relative abundances of *Faecalibacterium* and *Coproccus* bacteria were associated with higher quality of life, and a reduction of *Coproccus* and *Dialister* spp were linked to depression, an observation that was validated in a second cohort [43]. These population-level findings have been reproduced in a recent clinical study of female depressed individuals compared to healthy volunteers; this study used random forest models to identify bacterial genera that are enriched in healthy individuals compared to depressed individuals, which included *Faecalibacterium* and *Coprococcus* [16]. Several other studies comparing healthy volunteers to depressed individuals have reported reduced abundance of *Faecalibacterium* in depressed individuals and reduced abundance of members of the *Rumminococcaceae* family [11, 13, 16, 65, 66]. *Faecalibacterium prausnitzii* is an abundant commensal in the healthy human gut, plays an important role in gut physiology [67, 68], and is a key butyrate producer similar to the hub taxa associated with anxiety and depressive symptoms in the current study. A recent study also identified bacterial genera enriched in depression including *Escherichia-Shigella* and *Alistipes*, taxa that are suggested to be associated with increased inflammation [16], supporting a role for microbe-immune signaling in depression.

Overall, the analysis of 179 current or formally depressed individuals presented here demonstrates the benefit of utilizing microbiome analysis to better understand the clinical heterogeneity in depression. The results of this study should be considered preliminary as replication of our findings in independent samples is required to validate the clinically relevant module, to verify the clinical utility of identified hub taxa, and to determine the generalizability of the results to a broad population of depressed patients. There are some limitations to these analyses: participants were recruited from a limited geographical region (Texas, US); the analysis was cross-sectional and did not consider factors that influence microbiota composition and function, such as diet and exercise; and the lack of a healthy control cohort limits inference of the results to normal microbe-host interactions.

In summary, this study identified three co-occurrence bacterial modules using 16S microbiome data from a broad sample of depressed participants. One of these co-occurrence modules was significantly associated with clinical depression and anxiety. Based on our results we propose that reduced abundance of butyrate-producing taxa and increased abundance of inflammatory-related taxa may drive increased anxiety and depressive symptoms in depression. An important feature of this work was the innovative, data-driven approach that integrated both the compositional nature of the microbiome, as well as the community structure. These results are a critical step toward understanding the association between the microbiome and depression, and how a community-driven approach may facilitate effective precision medicine to improve clinical outcome.

## Supplementary information

Supplemental Tables

Supplemental Figures

## Data Availability

Data available upon request.

## Code availability

The retain resolve agglomeration algorithm has been developed into a tool available in the retain-resolve package: https://github.com/SarahAsbury/retainresolve. R scripts used in these analyses are available in the following repository: https://github.com/SarahAsbury/D2K_p1.
