# Supplementary material for: Leveraging the microbiome to understand clinical heterogeneity in depression: findings from the T-RAD study
Source: Transl Psychiatry. 2023 Apr 28;13:139. doi: 10.1038/s41398-023-02416-3 (PMC10147668; doi:10.1038/s41398-023-02416-3)
Supplement: Supplementary file 1 — Supplemental Tables [file 41398_2023_2416_MOESM1_ESM.pdf]

Supplementary Table 1: Retain-resolved taxa full taxonomy and resolution reference key with population statistics. Each taxa is assignd a unique ID (spID) To differentiate unique ASVs or agglomerated genus-level taxa with equivalent taxonomy,.

Taxa Full Label	Level	Statistics		spID	Taxonomy					
		Prevalence	Mean Relative Abundance		Kingdom	Phylum	Class	Order	Family	Genus
sp1_Bacteria_Firmicutes_Clostridia_Clostridiales_Lachnospiraceae_Blautia	ASV	0.96	5.2E-02	sp1	Bacteria	Firmicutes	Clostridia	Clostridiales	Lachnospiraceae	Blautia
sp2_Bacteria_Firmicutes_Clostridia_Clostridiales_Lachnospiraceae_Agathobacter	ASV	0.79	3.9E-02	sp2	Bacteria	Firmicutes	Clostridia	Clostridiales	Lachnospiraceae	Agathobacter
sp3_Bacteria_Bacteroidetes_Bacteroidia_Bacteroidales_Bacteroidaceae_Bacteroides	ASV	0.78	2.1E-02	sp3	Bacteria	Bacteroidetes	Bacteroidia	Bacteroidales	Bacteroidaceae	Bacteroides
sp4_Bacteria_Firmicutes_Clostridia_Clostridiales_Ruminococcaceae_Faecalibacterium	ASV	0.75	1.7E-02	sp4	Bacteria	Firmicutes	Clostridia	Clostridiales	Ruminococcaceae	Faecalibacterium
sp5_Bacteria_Firmicutes_Clostridia_Clostridiales_Ruminococcaceae_Faecalibacterium	ASV	0.75	1.5E-02	sp5	Bacteria	Firmicutes	Clostridia	Clostridiales	Ruminococcaceae	Faecalibacterium
sp6_Bacteria_Firmicutes_Clostridia_Clostridiales_Lachnospiraceae_NA	ASV	0.80	1.6E-02	sp6	Bacteria	Firmicutes	Clostridia	Clostridiales	Lachnospiraceae	NA
sp7_Bacteria_Bacteroidetes_Bacteroidia_Bacteroidales_Bacteroidaceae_Bacteroides	ASV	0.50	9.6E-03	sp7	Bacteria	Bacteroidetes	Bacteroidia	Bacteroidales	Bacteroidaceae	Bacteroides
sp8_Bacteria_Bacteroidetes_Bacteroidia_Bacteroidales_Bacteroidaceae_Bacteroides	ASV	0.56	1.2E-02	sp8	Bacteria	Bacteroidetes	Bacteroidia	Bacteroidales	Bacteroidaceae	Bacteroides
sp9_Bacteria_Bacteroidetes_Bacteroidia_Bacteroidales_Bacteroidaceae_Bacteroides	ASV	0.49	1.1E-02	sp9	Bacteria	Bacteroidetes	Bacteroidia	Bacteroidales	Bacteroidaceae	Bacteroides
sp10_Bacteria_Firmicutes_Clostridia_Clostridiales_Lachnospiraceae_Fusicatenibacter	ASV	0.85	1.3E-02	sp10	Bacteria	Firmicutes	Clostridia	Clostridiales	Lachnospiraceae	Fusicatenibacter
sp11_Bacteria_Firmicutes_Clostridia_Clostridiales_Ruminococcaceae_Subdoligranulum	ASV	0.72	1.7E-02	sp11	Bacteria	Firmicutes	Clostridia	Clostridiales	Ruminococcaceae	Subdoligranulum
sp12_Bacteria_Proteobacteria_Gammaproteobacteria_Enterobacteriales_Enterobacteriaceae_Escherichia/Shigella	ASV	0.71	9.4E-03	sp12	Bacteria	Proteobacteria	Gammaproteobacteria	Enterobacteriales	Enterobacteriaceae	Escherichia/Shigella
sp13_Bacteria_Firmicutes_Clostridia_Clostridiales_Lachnospiraceae_Dorea	ASV	0.65	1.3E-02	sp13	Bacteria	Firmicutes	Clostridia	Clostridiales	Lachnospiraceae	Dorea
sp14_Bacteria_Bacteroidetes_Bacteroidia_Bacteroidales_Bacteroidaceae_Bacteroides	ASV	0.39	1.1E-02	sp14	Bacteria	Bacteroidetes	Bacteroidia	Bacteroidales	Bacteroidaceae	Bacteroides
sp15_Bacteria_Firmicutes_Clostridia_Clostridiales_Ruminococcaceae_Faecalibacterium	ASV	0.56	9.9E-03	sp15	Bacteria	Firmicutes	Clostridia	Clostridiales	Ruminococcaceae	Faecalibacterium
sp16_Bacteria_Firmicutes_Clostridia_Clostridiales_Lachnospiraceae_Anaerostipes	ASV	0.87	1.0E-02	sp16	Bacteria	Firmicutes	Clostridia	Clostridiales	Lachnospiraceae	Anaerostipes
sp17_Bacteria_Bacteroidetes_Bacteroidia_Bacteroidales_Bacteroidaceae_Bacteroides	ASV	0.29	1.1E-02	sp17	Bacteria	Bacteroidetes	Bacteroidia	Bacteroidales	Bacteroidaceae	Bacteroides
sp18_Bacteria_Verrucomicrobia_Verrucomicrobiae_Verrucomicrobiales_Akkermansiaceae_Akkermansia	ASV	0.30	9.6E-03	sp18	Bacteria	Verrucomicrobia	Verrucomicrobiae	Verrucomicrobiales	Akkermansiaceae	Akkermansia
sp19_Bacteria_Firmicutes_Clostridia_Clostridiales_Lachnospiraceae_Blautia	ASV	0.78	7.9E-03	sp19	Bacteria	Firmicutes	Clostridia	Clostridiales	Lachnospiraceae	Blautia
sp20_Bacteria_Firmicutes_Clostridia_Clostridiales_Ruminococcaceae_Subdoligranulum	ASV	0.66	9.2E-03	sp20	Bacteria	Firmicutes	Clostridia	Clostridiales	Ruminococcaceae	Subdoligranulum
sp21_Bacteria_Firmicutes_Clostridia_Clostridiales_Lachnospiraceae_Blautia	ASV	0.77	9.8E-03	sp21	Bacteria	Firmicutes	Clostridia	Clostridiales	Lachnospiraceae	Blautia
sp22_Bacteria_Firmicutes_Clostridia_Clostridiales_Lachnospiraceae_Blautia	ASV	0.83	9.0E-03	sp22	Bacteria	Firmicutes	Clostridia	Clostridiales	Lachnospiraceae	Blautia
sp23_Bacteria_Bacteroidetes_Bacteroidia_Bacteroidales_Tannerellaceae_Parabacteroides	ASV	0.66	7.5E-03	sp23	Bacteria	Bacteroidetes	Bacteroidia	Bacteroidales	Tannerellaceae	Parabacteroides
sp24_Bacteria_Firmicutes_Clostridia_Clostridiales_Lachnospiraceae_NA	ASV	0.63	9.3E-03	sp24	Bacteria	Firmicutes	Clostridia	Clostridiales	Lachnospiraceae	NA
sp25_Bacteria_Firmicutes_Clostridia_Clostridiales_Ruminococcaceae_Faecalibacterium	ASV	0.48	9.1E-03	sp25	Bacteria	Firmicutes	Clostridia	Clostridiales	Ruminococcaceae	Faecalibacterium
sp26_Bacteria_Firmicutes_Clostridia_Clostridiales_Lachnospiraceae_Roseburia	ASV	0.34	7.5E-03	sp26	Bacteria	Firmicutes	Clostridia	Clostridiales	Lachnospiraceae	Roseburia
sp27_Bacteria_Bacteroidetes_Bacteroidia_Bacteroidales_Rikenellaceae_Alistipes	ASV	0.52	5.6E-03	sp27	Bacteria	Bacteroidetes	Bacteroidia	Bacteroidales	Rikenellaceae	Alistipes
sp28_Bacteria_Firmicutes_Clostridia_Clostridiales_Lachnospiraceae_NA	ASV	0.53	7.0E-03	sp28	Bacteria	Firmicutes	Clostridia	Clostridiales	Lachnospiraceae	NA
sp29_Bacteria_Firmicutes_Clostridia_Clostridiales_Lachnospiraceae_NA	ASV	0.74	6.8E-03	sp29	Bacteria	Firmicutes	Clostridia	Clostridiales	Lachnospiraceae	NA
sp30_Bacteria_Bacteroidetes_Bacteroidia_Bacteroidales_Rikenellaceae_Alistipes	ASV	0.59	6.4E-03	sp30	Bacteria	Bacteroidetes	Bacteroidia	Bacteroidales	Rikenellaceae	Alistipes
sp31_Bacteria_Bacteroidetes_Bacteroidia_Bacteroidales_Bacteroidaceae_Bacteroides	ASV	0.66	6.7E-03	sp31	Bacteria	Bacteroidetes	Bacteroidia	Bacteroidales	Bacteroidaceae	Bacteroides
sp32_Bacteria_Bacteroidetes_Bacteroidia_Bacteroidales_Bacteroidaceae_Bacteroides	ASV	0.27	4.0E-03	sp32	Bacteria	Bacteroidetes	Bacteroidia	Bacteroidales	Bacteroidaceae	Bacteroides
sp33_Bacteria_Firmicutes_Clostridia_Clostridiales_Ruminococcaceae_Ruminococcus_2	ASV	0.23	5.6E-03	sp33	Bacteria	Firmicutes	Clostridia	Clostridiales	Ruminococcaceae	Ruminococcus_2
sp34_Bacteria_Firmicutes_Clostridia_Clostridiales_Lachnospiraceae_Blautia	ASV	0.53	6.7E-03	sp34	Bacteria	Firmicutes	Clostridia	Clostridiales	Lachnospiraceae	Blautia
sp35_Bacteria_Bacteroidetes_Bacteroidia_Bacteroidales_Bacteroidaceae_Bacteroides	ASV	0.30	6.2E-03	sp35	Bacteria	Bacteroidetes	Bacteroidia	Bacteroidales	Bacteroidaceae	Bacteroides
sp36_Bacteria_Bacteroidetes_Bacteroidia_Bacteroidales_Bacteroidaceae_Bacteroides	ASV	0.45	4.3E-03	sp36	Bacteria	Bacteroidetes	Bacteroidia	Bacteroidales	Bacteroidaceae	Bacteroides
sp37_Bacteria_Firmicutes_Erysipelotrichia_Erysipelotrichales_Erysipelotrichaceae_Erysipelotrichaceae_UCG-003	ASV	0.44	6.1E-03	sp37	Bacteria	Firmicutes	Erysipelotrichia	Erysipelotrichales	Erysipelotrichaceae	Erysipelotrichaceae_UCG-003
sp38_Bacteria_Firmicutes_Negativicutes_Selenomonadales_Veillonellaceae_Dialister	ASV	0.41	5.1E-03	sp38	Bacteria	Firmicutes	Negativicutes	Selenomonadales	Veillonellaceae	Dialister
sp39_Bacteria_Firmicutes_Clostridia_Clostridiales_Ruminococcaceae_Ruminococcus_2	ASV	0.21	5.9E-03	sp39	Bacteria	Firmicutes	Clostridia	Clostridiales	Ruminococcaceae	Ruminococcus_2
sp40_Bacteria_Firmicutes_Clostridia_Clostridiales_Lachnospiraceae_NA	ASV	0.50	4.9E-03	sp40	Bacteria	Firmicutes	Clostridia	Clostridiales	Lachnospiraceae	NA
sp41_Bacteria_Bacteroidetes_Bacteroidia_Bacteroidales_Bacteroidaceae_Bacteroides	ASV	0.26	4.6E-03	sp41	Bacteria	Bacteroidetes	Bacteroidia	Bacteroidales	Bacteroidaceae	Bacteroides
sp42_Bacteria_Firmicutes_Bacilli_Lactobacillales_Streptococcaceae_Streptococcus	ASV	0.69	3.7E-03	sp42	Bacteria	Firmicutes	Bacilli	Lactobacillales	Streptococcaceae	Streptococcus
sp43_Bacteria_Firmicutes_Clostridia_Clostridiales_Lachnospiraceae_Anaerostipes	ASV	0.68	5.3E-03	sp43	Bacteria	Firmicutes	Clostridia	Clostridiales	Lachnospiraceae	Anaerostipes
sp44_Bacteria_Firmicutes_Clostridia_Clostridiales_Ruminococcaceae_Ruminococcaceae_UCG-002	ASV	0.39	4.3E-03	sp44	Bacteria	Firmicutes	Clostridia	Clostridiales	Ruminococcaceae	Ruminococcaceae_UCG-002
sp45_Bacteria_Firmicutes_Clostridia_Clostridiales_Lachnospiraceae_Roseburia	ASV	0.39	4.3E-03	sp45	Bacteria	Firmicutes	Clostridia	Clostridiales	Lachnospiraceae	Roseburia
sp46_Bacteria_Firmicutes_Clostridia_Clostridiales_Peptostreptococcaceae_Intestinibacter	ASV	0.58	4.7E-03	sp46	Bacteria	Firmicutes	Clostridia	Clostridiales	Peptostreptococcaceae	Intestinibacter
sp47_Bacteria_Firmicutes_Clostridia_Clostridiales_Lachnospiraceae_Lachnospiraceae_NK4A136_group	ASV	0.40	3.1E-03	sp47	Bacteria	Firmicutes	Clostridia	Clostridiales	Lachnospiraceae	Lachnospiraceae_NK4A136_group
sp48_Bacteria_Firmicutes_Clostridia_Clostridiales_Ruminococcaceae_Ruminiclostridium_5	ASV	0.57	4.7E-03	sp48	Bacteria	Firmicutes	Clostridia	Clostridiales	Ruminococcaceae	Ruminiclostridium_5
sp49_Bacteria_Bacteroidetes_Bacteroidia_Bacteroidales_Bacteroidaceae_Bacteroides	ASV	0.28	2.9E-03	sp49	Bacteria	Bacteroidetes	Bacteroidia	Bacteroidales	Bacteroidaceae	Bacteroides
sp50_Bacteria_Verrucomicrobia_Verrucomicrobiae_Verrucomicrobiales_Akkermansiaceae_Akkermansia	ASV	0.17	3.8E-03	sp50	Bacteria	Verrucomicrobia	Verrucomicrobiae	Verrucomicrobiales	Akkermansiaceae	Akkermansia
sp51_Bacteria_Firmicutes_Clostridia_Clostridiales_Lachnospiraceae_Roseburia	ASV	0.33	3.7E-03	sp51	Bacteria	Firmicutes	Clostridia	Clostridiales	Lachnospiraceae	Roseburia
sp52_Bacteria_Firmicutes_Clostridia_Clostridiales_Lachnospiraceae_Coprococcus_3	ASV	0.42	3.4E-03	sp52	Bacteria	Firmicutes	Clostridia	Clostridiales	Lachnospiraceae	Coprococcus_3
sp53_Bacteria_Firmicutes_Clostridia_Clostridiales_Lachnospiraceae_Lachnoclostridium	ASV	0.72	2.7E-03	sp53	Bacteria	Firmicutes	Clostridia	Clostridiales	Lachnospiraceae	Lachnoclostridium
sp54_Bacteria_Firmicutes_Clostridia_Clostridiales_Lachnospiraceae_NA	ASV	0.45	3.7E-03	sp54	Bacteria	Firmicutes	Clostridia	Clostridiales	Lachnospiraceae	NA
sp55_Bacteria_Actinobacteria_Actinobacteria_Bifidobacteriales_Bifidobacteriaceae_Bifidobacterium	ASV	0.26	5.4E-03	sp55	Bacteria	Actinobacteria	Actinobacteria	Bifidobacteriales	Bifidobacteriaceae	Bifidobacterium
sp56_Bacteria_Firmicutes_Clostridia_Clostridiales_Lachnospiraceae_Agathobacter	ASV	0.34	4.2E-03	sp56	Bacteria	Firmicutes	Clostridia	Clostridiales	Lachnospiraceae	Agathobacter
sp57_Bacteria_Bacteroidetes_Bacteroidia_Bacteroidales_Bacteroidaceae_Bacteroides	ASV	0.49	3.0E-03	sp57	Bacteria	Bacteroidetes	Bacteroidia	Bacteroidales	Bacteroidaceae	Bacteroides
sp58_Bacteria_Bacteroidetes_Bacteroidia_Bacteroidales_Bacteroidaceae_Bacteroides	ASV	0.21	3.5E-03	sp58	Bacteria	Bacteroidetes	Bacteroidia	Bacteroidales	Bacteroidaceae	Bacteroides
sp59_Bacteria_Bacteroidetes_Bacteroidia_Bacteroidales_Bacteroidaceae_Bacteroides	ASV	0.42	3.0E-03	sp59	Bacteria	Bacteroidetes	Bacteroidia	Bacteroidales	Bacteroidaceae	Bacteroides
sp60_Bacteria_Bacteroidetes_Bacteroidia_Bacteroidales_Bacteroidaceae_Bacteroides	ASV	0.11	1.5E-03	sp60	Bacteria	Bacteroidetes	Bacteroidia	Bacteroidales	Bacteroidaceae	Bacteroides
sp61_Bacteria_Firmicutes_Clostridia_Clostridiales_Lachnospiraceae_Dorea	ASV	0.68	3.6E-03	sp61	Bacteria	Firmicutes	Clostridia	Clostridiales	Lachnospiraceae	Dorea
sp62_Bacteria_Actinobacteria_Actinobacteria_Bifidobacteriales_Bifidobacteriaceae_Bifidobacterium	ASV	0.47	3.8E-03	sp62	Bacteria	Actinobacteria	Actinobacteria	Bifidobacteriales	Bifidobacteriaceae	Bifidobacterium
sp63_Bacteria_Firmicutes_Clostridia_Clostridiales_Ruminococcaceae_Ruminococcus_2	ASV	0.14	3.2E-03	sp63	Bacteria	Firmicutes	Clostridia	Clostridiales	Ruminococcaceae	Ruminococcus_2
sp64_Bacteria_Firmicutes_Erysipelotrichia_Erysipelotrichales_Erysipelotrichaceae_Erysipelotrichaceae_UCG-003	ASV	0.17	3.3E-03	sp64	Bacteria	Firmicutes	Erysipelotrichia	Erysipelotrichales	Erysipelotrichaceae	Erysipelotrichaceae_UCG-003
sp65_Bacteria_Bacteroidetes_Bacteroidia_Bacteroidales_Bacteroidaceae_Bacteroides	ASV	0.19	4.8E-03	sp65	Bacteria	Bacteroidetes	Bacteroidia	Bacteroidales	Bacteroidaceae	Bacteroides
sp66_Bacteria_Firmicutes_Clostridia_Clostridiales_Lachnospiraceae_Blautia	ASV	0.46	3.4E-03	sp66	Bacteria	Firmicutes	Clostridia	Clostridiales	Lachnospiraceae	Blautia
sp69_Bacteria_Bacteroidetes_Bacteroidia_Bacteroidales_Bacteroidaceae_Bacteroides	ASV	0.25	2.4E-03	sp69	Bacteria	Bacteroidetes	Bacteroidia	Bacteroidales	Bacteroidaceae	Bacteroides
sp70_Bacteria_Firmicutes_Clostridia_Clostridiales_Lachnospiraceae_Lachnospiraceae_NK4A136_group	ASV	0.51	2.4E-03	sp70	Bacteria	Firmicutes	Clostridia	Clostridiales	Lachnospiraceae	Lachnospiraceae_NK4A136_group
sp71_Bacteria_Firmicutes_Clostridia_Clostridiales_Lachnospiraceae_Roseburia	ASV	0.58	3.6E-03	sp71	Bacteria	Firmicutes	Clostridia	Clostridiales	Lachnospiraceae	Roseburia
sp72_Bacteria_Bacteroidetes_Bacteroidia_Bacteroidales_Bacteroidaceae_Bacteroides	ASV	0.15	3.0E-03	sp72	Bacteria	Bacteroidetes	Bacteroidia	Bacteroidales	Bacteroidaceae	Bacteroides
sp73_Bacteria_Firmicutes_Clostridia_Clostridiales_Ruminococcaceae_UBA1819	ASV	0.94	3.6E-03	sp73	Bacteria	Firmicutes	Clostridia	Clostridiales	Ruminococcaceae	UBA1819

sp74_Bacteria_Firmicutes_Clostridia_Clostridiales_Ruminococcaceae_NA	ASV	0.30	2.8E-03	sp74	Bacteria	Firmicutes	Clostridia	Clostridiales	Ruminococcaceae	NA
sp75_Bacteria_Firmicutes_Clostridia_Clostridiales_Ruminococcaceae_Ruminococcaceae_UCG-013	ASV	0.77	2.7E-03	sp75	Bacteria	Firmicutes	Clostridia	Clostridiales	Ruminococcaceae	Ruminococcaceae_UCG-013
sp76_Bacteria_Firmicutes_Erysipelotrichia_Erysipelotrichales_Erysipelotrichaceae_Catenibacterium	ASV	0.13	3.5E-03	sp76	Bacteria	Firmicutes	Erysipelotrichia	Erysipelotrichales	Erysipelotrichaceae	Catenibacterium
sp77_Bacteria_Firmicutes_Clostridia_Clostridiales_Ruminococcaceae_NA	ASV	0.18	2.8E-03	sp77	Bacteria	Firmicutes	Clostridia	Clostridiales	Ruminococcaceae	NA
sp78_Bacteria_Firmicutes_Clostridia_Clostridiales_Ruminococcaceae_Ruminococcus_2	ASV	0.12	3.1E-03	sp78	Bacteria	Firmicutes	Clostridia	Clostridiales	Ruminococcaceae	Ruminococcus_2
sp79_Bacteria_Firmicutes_Clostridia_Clostridiales_Ruminococcaceae_Ruminococcaceae_UCG-002	ASV	0.21	2.3E-03	sp79	Bacteria	Firmicutes	Clostridia	Clostridiales	Ruminococcaceae	Ruminococcaceae_UCG-002
sp80_Bacteria_Bacteroidetes_Bacteroidia_Bacteroidales_Tannerellaceae_Parabacteroides	ASV	0.27	1.8E-03	sp80	Bacteria	Bacteroidetes	Bacteroidia	Bacteroidales	Tannerellaceae	Parabacteroides
sp81_Bacteria_Firmicutes_Clostridia_Clostridiales_Clostridiaceae_1_Clostridium_sensu_stricto_1	ASV	0.43	2.8E-03	sp81	Bacteria	Firmicutes	Clostridia	Clostridiales	Clostridiaceae_1	Clostridium_sensu_stricto_1
sp82_Bacteria_Firmicutes_Clostridia_Clostridiales_Ruminococcaceae_NA	ASV	0.16	2.1E-03	sp82	Bacteria	Firmicutes	Clostridia	Clostridiales	Ruminococcaceae	NA
sp83_Bacteria_Firmicutes_Clostridia_Clostridiales_Ruminococcaceae_Faecalibacterium	ASV	0.29	2.1E-03	sp83	Bacteria	Firmicutes	Clostridia	Clostridiales	Ruminococcaceae	Faecalibacterium
sp84_Bacteria_Firmicutes_Clostridia_Clostridiales_Lachnospiraceae_Tyzzerella	ASV	0.26	2.1E-03	sp84	Bacteria	Firmicutes	Clostridia	Clostridiales	Lachnospiraceae	Tyzzerella
sp85_Bacteria_Firmicutes_Bacilli_Lactobacillales_Streptococcaceae_Streptococcus	ASV	0.65	1.4E-03	sp85	Bacteria	Firmicutes	Bacilli	Lactobacillales	Streptococcaceae	Streptococcus
sp86_Bacteria_Firmicutes_Clostridia_Clostridiales_Lachnospiraceae_Lachnospiraceae_NK4A136_group	ASV	0.45	2.2E-03	sp86	Bacteria	Firmicutes	Clostridia	Clostridiales	Lachnospiraceae	Lachnospiraceae_NK4A136_group
sp87_Bacteria_Actinobacteria_Coriobacteriia_Coriobacteriales_Coriobacteriaceae_Collinsella	ASV	0.62	3.1E-03	sp87	Bacteria	Actinobacteria	Coriobacteriia	Coriobacteriales	Coriobacteriaceae	Collinsella
sp88_Bacteria_Verrucomicrobia_Verrucomicrobiae_Verrucomicrobiales_Akkermansiaceae_Akkermansia	ASV	0.13	3.7E-03	sp88	Bacteria	Verrucomicrobia	Verrucomicrobiae	Verrucomicrobiales	Akkermansiaceae	Akkermansia
sp89_Bacteria_Bacteroidetes_Bacteroidia_Bacteroidales_Bacteroidaceae_Bacteroides	ASV	0.19	2.1E-03	sp89	Bacteria	Bacteroidetes	Bacteroidia	Bacteroidales	Bacteroidaceae	Bacteroides
sp90_Bacteria_Firmicutes_Clostridia_Clostridiales_Peptostreptococcaceae_Romboutsia	ASV	0.61	3.1E-03	sp90	Bacteria	Firmicutes	Clostridia	Clostridiales	Peptostreptococcaceae	Romboutsia
sp91_Bacteria_Actinobacteria_Actinobacteria_Bifidobacteriales_Bifidobacteriaceae_Bifidobacterium	ASV	0.30	2.8E-03	sp91	Bacteria	Actinobacteria	Actinobacteria	Bifidobacteriales	Bifidobacteriaceae	Bifidobacterium
sp93_Bacteria_Firmicutes_Clostridia_Clostridiales_Lachnospiraceae_NA	ASV	0.36	2.5E-03	sp93	Bacteria	Firmicutes	Clostridia	Clostridiales	Lachnospiraceae	NA
sp94_Bacteria_Firmicutes_Clostridia_Clostridiales_Ruminococcaceae_Ruminiclostridium_6	ASV	0.40	2.7E-03	sp94	Bacteria	Firmicutes	Clostridia	Clostridiales	Ruminococcaceae	Ruminiclostridium_6
sp95_Bacteria_Bacteroidetes_Bacteroidia_Bacteroidales_Bacteroidaceae_Bacteroides	ASV	0.11	1.7E-03	sp95	Bacteria	Bacteroidetes	Bacteroidia	Bacteroidales	Bacteroidaceae	Bacteroides
sp96_Bacteria_Firmicutes_Clostridia_Clostridiales_Ruminococcaceae_Ruminiclostridium_5	ASV	0.85	2.3E-03	sp96	Bacteria	Firmicutes	Clostridia	Clostridiales	Ruminococcaceae	Ruminiclostridium_5
sp97_Bacteria_Bacteroidetes_Bacteroidia_Bacteroidales_Bacteroidaceae_Bacteroides	ASV	0.36	1.8E-03	sp97	Bacteria	Bacteroidetes	Bacteroidia	Bacteroidales	Bacteroidaceae	Bacteroides
sp98_Bacteria_Bacteroidetes_Bacteroidia_Bacteroidales_Bacteroidaceae_Bacteroides	ASV	0.22	2.0E-03	sp98	Bacteria	Bacteroidetes	Bacteroidia	Bacteroidales	Bacteroidaceae	Bacteroides
sp100_Bacteria_Firmicutes_Negativicutes_Selenomonadales_Acidaminococcaceae_Acidaminococcus	ASV	0.21	1.7E-03	sp100	Bacteria	Firmicutes	Negativicutes	Selenomonadales	Acidaminococcaceae	Acidaminococcus
sp101_Bacteria_Firmicutes_Clostridia_Clostridiales_Lachnospiraceae_Lachnospiraceae_NK4A136_group	ASV	0.45	1.2E-03	sp101	Bacteria	Firmicutes	Clostridia	Clostridiales	Lachnospiraceae	Lachnospiraceae_NK4A136_group
sp102_Bacteria_Firmicutes_Clostridia_Clostridiales_Lachnospiraceae_NA	ASV	0.34	2.2E-03	sp102	Bacteria	Firmicutes	Clostridia	Clostridiales	Lachnospiraceae	NA
sp103_Bacteria_Bacteroidetes_Bacteroidia_Bacteroidales_Rikenellaceae_Alistipes	ASV	0.31	1.6E-03	sp103	Bacteria	Bacteroidetes	Bacteroidia	Bacteroidales	Rikenellaceae	Alistipes
sp104_Bacteria_Firmicutes_Bacilli_Lactobacillales_Streptococcaceae_Streptococcus	ASV	0.39	1.5E-03	sp104	Bacteria	Firmicutes	Bacilli	Lactobacillales	Streptococcaceae	Streptococcus
sp105_Bacteria_Firmicutes_Clostridia_Clostridiales_Ruminococcaceae_Ruminococcaceae_UCG-005	ASV	0.36	2.3E-03	sp105	Bacteria	Firmicutes	Clostridia	Clostridiales	Ruminococcaceae	Ruminococcaceae_UCG-005
sp106_Bacteria_Firmicutes_Clostridia_Clostridiales_Ruminococcaceae_Faecalibacterium	ASV	0.31	2.0E-03	sp106	Bacteria	Firmicutes	Clostridia	Clostridiales	Ruminococcaceae	Faecalibacterium
sp107_Bacteria_Firmicutes_Clostridia_Clostridiales_Ruminococcaceae_Ruminococcaceae_UCG-002	ASV	0.27	1.5E-03	sp107	Bacteria	Firmicutes	Clostridia	Clostridiales	Ruminococcaceae	Ruminococcaceae_UCG-002
sp109_Bacteria_Firmicutes_Erysipelotrichia_Erysipelotrichales_Erysipelotrichaceae_Faecalitalea	ASV	0.36	2.2E-03	sp109	Bacteria	Firmicutes	Erysipelotrichia	Erysipelotrichales	Erysipelotrichaceae	Faecalitalea
sp110_Bacteria_Firmicutes_Erysipelotrichia_Erysipelotrichales_Erysipelotrichaceae_Erysipelatoclostridium	ASV	0.58	1.5E-03	sp110	Bacteria	Firmicutes	Erysipelotrichia	Erysipelotrichales	Erysipelotrichaceae	Erysipelatoclostridium
sp111_Bacteria_Firmicutes_Clostridia_Clostridiales_Ruminococcaceae_Ruminococcus_1	ASV	0.21	1.2E-03	sp111	Bacteria	Firmicutes	Clostridia	Clostridiales	Ruminococcaceae	Ruminococcus_1
sp112_Bacteria_Firmicutes_Clostridia_Clostridiales_Ruminococcaceae_Ruminococcaceae_NK4A214_group	ASV	0.33	1.6E-03	sp112	Bacteria	Firmicutes	Clostridia	Clostridiales	Ruminococcaceae	Ruminococcaceae_NK4A214_group
sp114_Bacteria_Bacteroidetes_Bacteroidia_Bacteroidales_Bacteroidaceae_Bacteroides	ASV	0.26	1.6E-03	sp114	Bacteria	Bacteroidetes	Bacteroidia	Bacteroidales	Bacteroidaceae	Bacteroides
sp115_Bacteria_Firmicutes_Clostridia_Clostridiales_Ruminococcaceae_Ruminococcaceae_UCG-004	ASV	0.63	1.7E-03	sp115	Bacteria	Firmicutes	Clostridia	Clostridiales	Ruminococcaceae	Ruminococcaceae_UCG-004
sp116_Bacteria_Firmicutes_Clostridia_Clostridiales_Ruminococcaceae_Negativibacillus	ASV	0.36	1.5E-03	sp116	Bacteria	Firmicutes	Clostridia	Clostridiales	Ruminococcaceae	Negativibacillus
sp118_Bacteria_Bacteroidetes_Bacteroidia_Bacteroidales_Bacteroidaceae_Bacteroides	ASV	0.15	1.3E-03	sp118	Bacteria	Bacteroidetes	Bacteroidia	Bacteroidales	Bacteroidaceae	Bacteroides
sp119_Bacteria_Firmicutes_Clostridia_Clostridiales_Lachnospiraceae_Agathobacter	ASV	0.40	1.4E-03	sp119	Bacteria	Firmicutes	Clostridia	Clostridiales	Lachnospiraceae	Agathobacter
sp120_Bacteria_Firmicutes_Clostridia_Clostridiales_Lachnospiraceae_Roseburia	ASV	0.17	1.3E-03	sp120	Bacteria	Firmicutes	Clostridia	Clostridiales	Lachnospiraceae	Roseburia
sp121_Bacteria_Firmicutes_Clostridia_Clostridiales_Lachnospiraceae_Coprococcus_1	ASV	0.53	1.7E-03	sp121	Bacteria	Firmicutes	Clostridia	Clostridiales	Lachnospiraceae	Coprococcus_1
sp122_Bacteria_Firmicutes_Clostridia_Clostridiales_Lachnospiraceae_Lachnoclostridium	ASV	0.42	1.3E-03	sp122	Bacteria	Firmicutes	Clostridia	Clostridiales	Lachnospiraceae	Lachnoclostridium
sp123_Bacteria_Firmicutes_Clostridia_Clostridiales_Ruminococcaceae_Ruminococcaceae_UCG-002	ASV	0.42	1.4E-03	sp123	Bacteria	Firmicutes	Clostridia	Clostridiales	Ruminococcaceae	Ruminococcaceae_UCG-002
sp124_Bacteria_Firmicutes_Clostridia_Clostridiales_Lachnospiraceae_Lachnoclostridium	ASV	0.26	1.7E-03	sp124	Bacteria	Firmicutes	Clostridia	Clostridiales	Lachnospiraceae	Lachnoclostridium
sp125_Bacteria_Bacteroidetes_Bacteroidia_Bacteroidales_Tannerellaceae_Parabacteroides	ASV	0.17	1.3E-03	sp125	Bacteria	Bacteroidetes	Bacteroidia	Bacteroidales	Tannerellaceae	Parabacteroides
sp126_Bacteria_Firmicutes_Clostridia_Clostridiales_Ruminococcaceae_NA	ASV	0.14	1.4E-03	sp126	Bacteria	Firmicutes	Clostridia	Clostridiales	Ruminococcaceae	NA
sp127_Bacteria_Firmicutes_Clostridia_Clostridiales_Ruminococcaceae_Ruminococcaceae_UCG-013	ASV	0.40	1.7E-03	sp127	Bacteria	Firmicutes	Clostridia	Clostridiales	Ruminococcaceae	Ruminococcaceae_UCG-013
sp128_Bacteria_Bacteroidetes_Bacteroidia_Bacteroidales_Rikenellaceae_Alistipes	ASV	0.28	1.8E-03	sp128	Bacteria	Bacteroidetes	Bacteroidia	Bacteroidales	Rikenellaceae	Alistipes
sp129_Bacteria_Firmicutes_Clostridia_Clostridiales_Lachnospiraceae_Lachnoclostridium	ASV	0.47	1.1E-03	sp129	Bacteria	Firmicutes	Clostridia	Clostridiales	Lachnospiraceae	Lachnoclostridium
sp131_Bacteria_Firmicutes_Clostridia_Clostridiales_Lachnospiraceae_Anaerostipes	ASV	0.50	1.8E-03	sp131	Bacteria	Firmicutes	Clostridia	Clostridiales	Lachnospiraceae	Anaerostipes
sp134_Bacteria_Firmicutes_Clostridia_Clostridiales_Lachnospiraceae_NA	ASV	0.36	1.2E-03	sp134	Bacteria	Firmicutes	Clostridia	Clostridiales	Lachnospiraceae	NA
sp136_Bacteria_Firmicutes_Clostridia_Clostridiales_Lachnospiraceae_Anaerostipes	ASV	0.31	2.1E-03	sp136	Bacteria	Firmicutes	Clostridia	Clostridiales	Lachnospiraceae	Anaerostipes
sp137_Bacteria_Firmicutes_Clostridia_Clostridiales_Ruminococcaceae_Oscillibacter	ASV	0.70	1.5E-03	sp137	Bacteria	Firmicutes	Clostridia	Clostridiales	Ruminococcaceae	Oscillibacter
sp139_Bacteria_Firmicutes_Clostridia_Clostridiales_Lachnospiraceae_Lachnospira	ASV	0.41	1.2E-03	sp139	Bacteria	Firmicutes	Clostridia	Clostridiales	Lachnospiraceae	Lachnospira
sp140_Bacteria_Firmicutes_Erysipelotrichia_Erysipelotrichales_Erysipelotrichaceae_Turicibacter	ASV	0.44	1.5E-03	sp140	Bacteria	Firmicutes	Erysipelotrichia	Erysipelotrichales	Erysipelotrichaceae	Turicibacter
sp141_Bacteria_Bacteroidetes_Bacteroidia_Bacteroidales_Bacteroidaceae_Bacteroides	ASV	0.16	2.0E-03	sp141	Bacteria	Bacteroidetes	Bacteroidia	Bacteroidales	Bacteroidaceae	Bacteroides
sp142_Bacteria_Firmicutes_Clostridia_Clostridiales_Lachnospiraceae_Blautia	ASV	0.18	1.3E-03	sp142	Bacteria	Firmicutes	Clostridia	Clostridiales	Lachnospiraceae	Blautia
sp146_Bacteria_Firmicutes_Clostridia_Clostridiales_Ruminococcaceae_Butyricicoccus	ASV	0.58	1.4E-03	sp146	Bacteria	Firmicutes	Clostridia	Clostridiales	Ruminococcaceae	Butyricicoccus
sp149_Bacteria_Firmicutes_Clostridia_Clostridiales_Ruminococcaceae_Flavonifractor	ASV	0.68	1.4E-03	sp149	Bacteria	Firmicutes	Clostridia	Clostridiales	Ruminococcaceae	Flavonifractor
sp150_Bacteria_Firmicutes_Clostridia_Clostridiales_Lachnospiraceae_Lachnoclostridium	ASV	0.20	1.2E-03	sp150	Bacteria	Firmicutes	Clostridia	Clostridiales	Lachnospiraceae	Lachnoclostridium
sp152_Bacteria_Firmicutes_Clostridia_Clostridiales_Lachnospiraceae_Blautia	ASV	0.20	1.6E-03	sp152	Bacteria	Firmicutes	Clostridia	Clostridiales	Lachnospiraceae	Blautia
sp153_Bacteria_Bacteroidetes_Bacteroidia_Bacteroidales_Tannerellaceae_Parabacteroides	ASV	0.17	1.1E-03	sp153	Bacteria	Bacteroidetes	Bacteroidia	Bacteroidales	Tannerellaceae	Parabacteroides
sp154_Bacteria_Firmicutes_Clostridia_Clostridiales_Lachnospiraceae_Blautia	ASV	0.25	1.5E-03	sp154	Bacteria	Firmicutes	Clostridia	Clostridiales	Lachnospiraceae	Blautia
sp155_Bacteria_Firmicutes_Clostridia_Clostridiales_Christensenellaceae_Christensenellaceae_R-7_group	ASV	0.15	2.0E-03	sp155	Bacteria	Firmicutes	Clostridia	Clostridiales	Christensenellaceae	Christensenellaceae_R-7_group
sp156_Bacteria_Firmicutes_Clostridia_Clostridiales_Ruminococcaceae_Ruminococcaceae_NK4A214_group	ASV	0.23	1.6E-03	sp156	Bacteria	Firmicutes	Clostridia	Clostridiales	Ruminococcaceae	Ruminococcaceae_NK4A214_group
sp158_Bacteria_Firmicutes_Clostridia_Clostridiales_Lachnospiraceae_NA	ASV	0.13	1.1E-03	sp158	Bacteria	Firmicutes	Clostridia	Clostridiales	Lachnospiraceae	NA
sp162_Bacteria_Firmicutes_Clostridia_Clostridiales_Lachnospiraceae_Lachnospira	ASV	0.39	1.2E-03	sp162	Bacteria	Firmicutes	Clostridia	Clostridiales	Lachnospiraceae	Lachnospira
sp163_Bacteria_Firmicutes_Clostridia_Clostridiales_Ruminococcaceae_Ruminococcaceae_NK4A214_group	ASV	0.23	1.2E-03	sp163	Bacteria	Firmicutes	Clostridia	Clostridiales	Ruminococcaceae	Ruminococcaceae_NK4A214_group
sp168_Bacteria_Firmicutes_Clostridia_Clostridiales_Lachnospiraceae_Tyzzerella	ASV	0.11	1.1E-03	sp168	Bacteria	Firmicutes	Clostridia	Clostridiales	Lachnospiraceae	Tyzzerella
sp169_Bacteria_Firmicutes_Clostridia_Clostridiales_Lachnospiraceae_Blautia	ASV	0.24	1.2E-03	sp169	Bacteria	Firmicutes	Clostridia	Clostridiales	Lachnospiraceae	Blautia
sp171_Bacteria_Firmicutes_Clostridia_Clostridiales_Lachnospiraceae_Lachnospira	ASV	0.37	1.2E-03	sp171	Bacteria	Firmicutes	Clostridia	Clostridiales	Lachnospiraceae	Lachnospira
sp172_Bacteria_Firmicutes_Clostridia_Clostridiales_Lachnospiraceae_Blautia	ASV	0.47	1.2E-03	sp172	Bacteria	Firmicutes	Clostridia	Clostridiales	Lachnospiraceae	Blautia
sp173_Bacteria_Firmicutes_Clostridia_Clostridiales_Ruminococcaceae_Ruminococcus_1	ASV	0.10	1.0E-03	sp173	Bacteria	Firmicutes	Clostridia	Clostridiales	Ruminococcaceae	Ruminococcus_1
sp174_Bacteria_Proteobacteria_Gammaproteobacteria_Betaproteobacteriales_Burkholderiaceae_Parasutterella	ASV	0.33	1.2E-03	sp174	Bacteria	Proteobacteria	Gammaproteobacteria	Betaproteobacteriales	Burkholderiaceae	Parasutterella
sp175_Bacteria_Firmicutes_Clostridia_Clostridiales_Lachnospiraceae_Roseburia	ASV	0.20	1.2E-03	sp175	Bacteria	Firmicutes	Clostridia	Clostridiales	Lachnospiraceae	Roseburia
sp177_Bacteria_Firmicutes_Clostridia_Clostridiales_Ruminococcaceae_Ruminiclostridium_5	ASV	0.66	1.0E-03	sp177	Bacteria	Firmicutes	Clostridia	Clostridiales	Ruminococcaceae	Ruminiclostridium_5

sp179_Bacteria_Firmicutes_Clostridia_Clostridiales_Christensenellaceae_Christensenellaceae_R-7_group	ASV	0.41	1.0E-03	sp179	Bacteria	Firmicutes	Clostridia	Clostridiales	Christensenellaceae	Christensenellaceae_R-7_group
sp180_Bacteria_Firmicutes_Clostridia_Clostridiales_Lachnospiraceae_Roseburia	ASV	0.10	1.3E-03	sp180	Bacteria	Firmicutes	Clostridia	Clostridiales	Lachnospiraceae	Roseburia
sp185_Bacteria_Bacteroidetes_Bacteroidia_Bacteroidales_Rikenellaceae_Alistipes	ASV	0.11	1.1E-03	sp185	Bacteria	Bacteroidetes	Bacteroidia	Bacteroidales	Rikenellaceae	Alistipes
sp194_Bacteria_Bacteroidetes_Bacteroidia_Bacteroidales_Bacteroidaceae_Bacteroides	ASV	0.13	1.0E-03	sp194	Bacteria	Bacteroidetes	Bacteroidia	Bacteroidales	Bacteroidaceae	Bacteroides
sp196_Bacteria_Firmicutes_Clostridia_Clostridiales_Clostridiaceae_1_Clostridium_sensu_stricto_1	ASV	0.23	1.1E-03	sp196	Bacteria	Firmicutes	Clostridia	Clostridiales	Clostridiaceae_1	Clostridium_sensu_stricto_1
sp197_Bacteria_Firmicutes_Clostridia_Clostridiales_Peptostreptococcaceae_Terrisporobacter	ASV	0.15	1.1E-03	sp197	Bacteria	Firmicutes	Clostridia	Clostridiales	Peptostreptococcaceae	Terrisporobacter
sp200_Bacteria_Firmicutes_Clostridia_Clostridiales_Lachnospiraceae_Lachnospiraceae_ND3007_group	ASV	0.54	8.9E-04	sp200	Bacteria	Firmicutes	Clostridia	Clostridiales	Lachnospiraceae	Lachnospiraceae_ND3007_group
sp201_Bacteria_Firmicutes_Clostridia_Clostridiales_Christensenellaceae_Christensenellaceae_R-7_group	ASV	0.14	1.0E-03	sp201	Bacteria	Firmicutes	Clostridia	Clostridiales	Christensenellaceae	Christensenellaceae_R-7_group
sp208_Bacteria_Firmicutes_Clostridia_Clostridiales_Ruminococcaceae_Ruminococcaceae_UCG-002	ASV	0.15	1.2E-03	sp208	Bacteria	Firmicutes	Clostridia	Clostridiales	Ruminococcaceae	Ruminococcaceae_UCG-002
sp217_Bacteria_Firmicutes_Clostridia_Clostridiales_Lachnospiraceae_Blautia	ASV	0.15	1.3E-03	sp217	Bacteria	Firmicutes	Clostridia	Clostridiales	Lachnospiraceae	Blautia
sp221_Bacteria_Firmicutes_Clostridia_Clostridiales_Ruminococcaceae_Ruminococcaceae_UCG-002	ASV	0.10	1.1E-03	sp221	Bacteria	Firmicutes	Clostridia	Clostridiales	Ruminococcaceae	Ruminococcaceae_UCG-002
sp231_Bacteria_Firmicutes_Clostridia_Clostridiales_Lachnospiraceae_Blautia	ASV	0.11	1.0E-03	sp231	Bacteria	Firmicutes	Clostridia	Clostridiales	Lachnospiraceae	Blautia
sp236_Bacteria_Proteobacteria_Deltaproteobacteria_Desulfovibrionales_Desulfovibrionaceae_Bilophila	ASV	0.57	6.4E-04	sp236	Bacteria	Proteobacteria	Deltaproteobacteria	Desulfovibrionales	Desulfovibrionaceae	Bilophila
sp240_Bacteria_Firmicutes_Clostridia_Clostridiales_Christensenellaceae_Christensenellaceae_R-7_group	ASV	0.26	1.0E-03	sp240	Bacteria	Firmicutes	Clostridia	Clostridiales	Christensenellaceae	Christensenellaceae_R-7_group
sp254_Bacteria_Firmicutes_Clostridia_Clostridiales_Family_XIII_Family_XIII_AD3011_group	ASV	0.50	5.7E-04	sp254	Bacteria	Firmicutes	Clostridia	Clostridiales	Family_XIII	Family_XIII_AD3011_group
sp375_Bacteria_Firmicutes_Erysipelotrichia_Erysipelotrichales_Erysipelotrichaceae_Holdemania	ASV	0.52	3.6E-04	sp375	Bacteria	Firmicutes	Erysipelotrichia	Erysipelotrichales	Erysipelotrichaceae	Holdemania
sp67_Bacteria_Firmicutes_Bacilli_Lactobacillales_Streptococcaceae_Streptococcus	genus	0.79	8.2E-03	sp67	Bacteria	Firmicutes	Bacilli	Lactobacillales	Streptococcaceae	Streptococcus
sp68_Bacteria_Bacteroidetes_Bacteroidia_Bacteroidales_Bacteroidaceae_Bacteroides	genus	0.95	4.0E-02	sp68	Bacteria	Bacteroidetes	Bacteroidia	Bacteroidales	Bacteroidaceae	Bacteroides
sp108_Bacteria_Firmicutes_Erysipelotrichia_Erysipelotrichales_Erysipelotrichaceae_Holdemanella	genus	0.15	3.6E-03	sp108	Bacteria	Firmicutes	Erysipelotrichia	Erysipelotrichales	Erysipelotrichaceae	Holdemanella
sp132_Bacteria_Firmicutes_Bacilli_Lactobacillales_Lactobacillaceae_Lactobacillus	genus	0.39	1.7E-03	sp132	Bacteria	Firmicutes	Bacilli	Lactobacillales	Lactobacillaceae	Lactobacillus
sp135_Bacteria_Proteobacteria_Gammaproteobacteria_Betaproteobacteriales_Burkholderiaceae_Parasutterella	genus	0.39	1.4E-03	sp135	Bacteria	Proteobacteria	Gammaproteobacteria	Betaproteobacteriales	Burkholderiaceae	Parasutterella
sp145_Bacteria_Firmicutes_Bacilli_Lactobacillales_Enterococcaceae_Enterococcus	genus	0.18	3.4E-04	sp145	Bacteria	Firmicutes	Bacilli	Lactobacillales	Enterococcaceae	Enterococcus
sp148_Bacteria_Bacteroidetes_Bacteroidia_Bacteroidales_Rikenellaceae_Alistipes	genus	0.73	6.5E-03	sp148	Bacteria	Bacteroidetes	Bacteroidia	Bacteroidales	Rikenellaceae	Alistipes
sp151_Bacteria_Firmicutes_Erysipelotrichia_Erysipelotrichales_Erysipelotrichaceae_Catenibacterium	genus	0.13	2.5E-03	sp151	Bacteria	Firmicutes	Erysipelotrichia	Erysipelotrichales	Erysipelotrichaceae	Catenibacterium
sp161_Bacteria_Firmicutes_Clostridia_Clostridiales_Christensenellaceae_Christensenellaceae_R-7_group	genus	0.61	8.7E-03	sp161	Bacteria	Firmicutes	Clostridia	Clostridiales	Christensenellaceae	Christensenellaceae_R-7_group
sp165_Bacteria_Firmicutes_Negativicutes_Selenomonadales_Veillonellaceae_Dialister	genus	0.39	3.0E-03	sp165	Bacteria	Firmicutes	Negativicutes	Selenomonadales	Veillonellaceae	Dialister
sp166_Bacteria_Proteobacteria_Gammaproteobacteria_Enterobacteriales_Enterobacteriaceae_Klebsiella	genus	0.17	2.9E-03	sp166	Bacteria	Proteobacteria	Gammaproteobacteria	Enterobacteriales	Enterobacteriaceae	Klebsiella
sp170_Bacteria_Firmicutes_Negativicutes_Selenomonadales_Acidaminococcaceae_Phascolarctobacterium	genus	0.51	1.7E-03	sp170	Bacteria	Firmicutes	Negativicutes	Selenomonadales	Acidaminococcaceae	Phascolarctobacterium
sp176_Bacteria_Fusobacteria_Fusobacteriia_Fusobacteriales_Fusobacteriaceae_Fusobacterium	genus	0.17	2.5E-03	sp176	Bacteria	Fusobacteria	Fusobacteriia	Fusobacteriales	Fusobacteriaceae	Fusobacterium
sp178_Bacteria_Firmicutes_Clostridia_Clostridiales_Lachnospiraceae_NA	genus	0.99	2.0E-02	sp178	Bacteria	Firmicutes	Clostridia	Clostridiales	Lachnospiraceae	NA
sp182_Bacteria_Firmicutes_Clostridia_Clostridiales_Lachnospiraceae_Lachnospiraceae_UCG-001	genus	0.47	1.3E-03	sp182	Bacteria	Firmicutes	Clostridia	Clostridiales	Lachnospiraceae	Lachnospiraceae_UCG-001
sp183_Bacteria_Firmicutes_Clostridia_Clostridiales_Lachnospiraceae_CAG-56	genus	0.34	1.1E-03	sp183	Bacteria	Firmicutes	Clostridia	Clostridiales	Lachnospiraceae	CAG-56
sp187_Bacteria_Firmicutes_Erysipelotrichia_Erysipelotrichales_Erysipelotrichaceae_Erysipelatoclostridium	genus	0.72	1.6E-03	sp187	Bacteria	Firmicutes	Erysipelotrichia	Erysipelotrichales	Erysipelotrichaceae	Erysipelatoclostridium
sp189_Bacteria_Firmicutes_Clostridia_Clostridiales_Lachnospiraceae_Roseburia	genus	0.73	4.5E-03	sp189	Bacteria	Firmicutes	Clostridia	Clostridiales	Lachnospiraceae	Roseburia
sp190_Bacteria_Firmicutes_Clostridia_Clostridiales_Lachnospiraceae_Blautia	genus	0.93	1.3E-02	sp190	Bacteria	Firmicutes	Clostridia	Clostridiales	Lachnospiraceae	Blautia
sp191_Bacteria_Bacteroidetes_Bacteroidia_Bacteroidales_Prevotellaceae_Prevotella_9	genus	0.20	7.8E-03	sp191	Bacteria	Bacteroidetes	Bacteroidia	Bacteroidales	Prevotellaceae	Prevotella_9
sp193_Bacteria_Firmicutes_Clostridia_Clostridiales_Ruminococcaceae_Ruminococcaceae_UCG-014	genus	0.42	7.0E-03	sp193	Bacteria	Firmicutes	Clostridia	Clostridiales	Ruminococcaceae	Ruminococcaceae_UCG-014
sp195_Bacteria_Firmicutes_Clostridia_Clostridiales_Lachnospiraceae_Lachnoclostridium	genus	0.98	9.7E-03	sp195	Bacteria	Firmicutes	Clostridia	Clostridiales	Lachnospiraceae	Lachnoclostridium
sp198_Bacteria_Bacteroidetes_Bacteroidia_Bacteroidales_Muribaculaceae_NA	genus	0.14	2.3E-03	sp198	Bacteria	Bacteroidetes	Bacteroidia	Bacteroidales	Muribaculaceae	NA
sp199_Bacteria_Firmicutes_Clostridia_Clostridiales_Ruminococcaceae_Ruminiclostridium_9	genus	0.85	1.7E-03	sp199	Bacteria	Firmicutes	Clostridia	Clostridiales	Ruminococcaceae	Ruminiclostridium_9
sp204_Bacteria_Firmicutes_Clostridia_Clostridiales_Ruminococcaceae_Ruminococcus_1	genus	0.58	4.5E-03	sp204	Bacteria	Firmicutes	Clostridia	Clostridiales	Ruminococcaceae	Ruminococcus_1
sp205_Bacteria_Firmicutes_Clostridia_Clostridiales_Lachnospiraceae_Anaerostipes	genus	0.75	3.8E-03	sp205	Bacteria	Firmicutes	Clostridia	Clostridiales	Lachnospiraceae	Anaerostipes
sp206_Bacteria_Firmicutes_Clostridia_Clostridiales_Ruminococcaceae_Ruminococcus_2	genus	0.19	4.0E-03	sp206	Bacteria	Firmicutes	Clostridia	Clostridiales	Ruminococcaceae	Ruminococcus_2
sp209_Bacteria_Firmicutes_Clostridia_Clostridiales_Ruminococcaceae_Faecalibacterium	genus	0.60	5.9E-03	sp209	Bacteria	Firmicutes	Clostridia	Clostridiales	Ruminococcaceae	Faecalibacterium
sp211_Bacteria_Firmicutes_Clostridia_Clostridiales_Ruminococcaceae_Ruminiclostridium_5	genus	0.80	2.8E-03	sp211	Bacteria	Firmicutes	Clostridia	Clostridiales	Ruminococcaceae	Ruminiclostridium_5
sp213_Bacteria_Firmicutes_Clostridia_Clostridiales_Lachnospiraceae_Coprococcus_3	genus	0.43	1.8E-03	sp213	Bacteria	Firmicutes	Clostridia	Clostridiales	Lachnospiraceae	Coprococcus_3
sp219_Bacteria_Bacteroidetes_Bacteroidia_Bacteroidales_Tannerellaceae_Parabacteroides	genus	0.73	6.5E-03	sp219	Bacteria	Bacteroidetes	Bacteroidia	Bacteroidales	Tannerellaceae	Parabacteroides
sp223_Bacteria_Firmicutes_Clostridia_Clostridiales_Ruminococcaceae_Anaerofilum	genus	0.48	8.5E-04	sp223	Bacteria	Firmicutes	Clostridia	Clostridiales	Ruminococcaceae	Anaerofilum
sp224_Bacteria_Firmicutes_Clostridia_Clostridiales_Ruminococcaceae_Ruminococcaceae_UCG-005	genus	0.74	2.4E-03	sp224	Bacteria	Firmicutes	Clostridia	Clostridiales	Ruminococcaceae	Ruminococcaceae_UCG-005
sp226_Bacteria_Firmicutes_Clostridia_Clostridiales_Family_XIII_Family_XIII_AD3011_group	genus	0.78	2.2E-03	sp226	Bacteria	Firmicutes	Clostridia	Clostridiales	Family_XIII	Family_XIII_AD3011_group
sp238_Bacteria_Firmicutes_Clostridia_Clostridiales_Ruminococcaceae_DTU089	genus	0.79	1.2E-03	sp238	Bacteria	Firmicutes	Clostridia	Clostridiales	Ruminococcaceae	DTU089
sp244_Bacteria_Actinobacteria_Actinobacteria_Bifidobacteriales_Bifidobacteriaceae_Bifidobacterium	genus	0.53	2.0E-03	sp244	Bacteria	Actinobacteria	Actinobacteria	Bifidobacteriales	Bifidobacteriaceae	Bifidobacterium
sp251_Bacteria_Firmicutes_Clostridia_Clostridiales_Lachnospiraceae_Coprococcus_2	genus	0.16	1.3E-03	sp251	Bacteria	Firmicutes	Clostridia	Clostridiales	Lachnospiraceae	Coprococcus_2
sp255_Bacteria_Firmicutes_Clostridia_Clostridiales_Ruminococcaceae_NA	genus	0.94	8.5E-03	sp255	Bacteria	Firmicutes	Clostridia	Clostridiales	Ruminococcaceae	NA
sp258_Bacteria_Firmicutes_Clostridia_Clostridiales_Lachnospiraceae_Lachnospiraceae_UCG-010	genus	0.83	1.7E-03	sp258	Bacteria	Firmicutes	Clostridia	Clostridiales	Lachnospiraceae	Lachnospiraceae_UCG-010
sp259_Bacteria_Firmicutes_Clostridia_Clostridiales_Ruminococcaceae_Ruminococcaceae_UCG-003	genus	0.47	8.2E-04	sp259	Bacteria	Firmicutes	Clostridia	Clostridiales	Ruminococcaceae	Ruminococcaceae_UCG-003
sp261_Bacteria_Firmicutes_Clostridia_Clostridiales_Lachnospiraceae_Tyzzerella_4	genus	0.25	2.0E-03	sp261	Bacteria	Firmicutes	Clostridia	Clostridiales	Lachnospiraceae	Tyzzerella_4
sp262_Bacteria_Firmicutes_Clostridia_Clostridiales_Family_XIII_Family_XIII_UCG-001	genus	0.60	6.4E-04	sp262	Bacteria	Firmicutes	Clostridia	Clostridiales	Family_XIII	Family_XIII_UCG-001
sp263_Bacteria_Firmicutes_Erysipelotrichia_Erysipelotrichales_Erysipelotrichaceae_Candidatus_Stoquefichus	genus	0.33	4.4E-04	sp263	Bacteria	Firmicutes	Erysipelotrichia	Erysipelotrichales	Erysipelotrichaceae	Candidatus_Stoquefichus
sp264_Bacteria_Firmicutes_Clostridia_Clostridiales_Ruminococcaceae_Flavonifractor	genus	0.62	9.1E-04	sp264	Bacteria	Firmicutes	Clostridia	Clostridiales	Ruminococcaceae	Flavonifractor
sp266_Bacteria_Firmicutes_Clostridia_Clostridiales_Lachnospiraceae_Lachnospira	genus	0.56	1.6E-03	sp266	Bacteria	Firmicutes	Clostridia	Clostridiales	Lachnospiraceae	Lachnospira
sp267_Bacteria_Firmicutes_Clostridia_Clostridiales_Lachnospiraceae_Sellimonas	genus	0.50	2.0E-03	sp267	Bacteria	Firmicutes	Clostridia	Clostridiales	Lachnospiraceae	Sellimonas
sp268_Bacteria_Proteobacteria_Deltaproteobacteria_Desulfovibrionales_Desulfovibrionaceae_Desulfovibrio	genus	0.35	1.9E-03	sp268	Bacteria	Proteobacteria	Deltaproteobacteria	Desulfovibrionales	Desulfovibrionaceae	Desulfovibrio
sp270_Bacteria_Firmicutes_Clostridia_Clostridiales_Lachnospiraceae_Lachnospiraceae_UCG-004	genus	0.41	7.4E-04	sp270	Bacteria	Firmicutes	Clostridia	Clostridiales	Lachnospiraceae	Lachnospiraceae_UCG-004
sp278_Bacteria_Firmicutes_Clostridia_Clostridiales_Family_XIII_Mogibacterium	genus	0.13	5.2E-04	sp278	Bacteria	Firmicutes	Clostridia	Clostridiales	Family_XIII	Mogibacterium
sp279_Bacteria_Bacteroidetes_Bacteroidia_Bacteroidales_Prevotellaceae_Paraprevotella	genus	0.18	1.0E-03	sp279	Bacteria	Bacteroidetes	Bacteroidia	Bacteroidales	Prevotellaceae	Paraprevotella
sp280_Bacteria_Firmicutes_Clostridia_Clostridiales_Ruminococcaceae_Ruminococcaceae_NK4A214_group	genus	0.36	2.6E-03	sp280	Bacteria	Firmicutes	Clostridia	Clostridiales	Ruminococcaceae	Ruminococcaceae_NK4A214_group
sp286_Bacteria_Firmicutes_Clostridia_Clostridiales_Ruminococcaceae_Pygmaiobacter	genus	0.48	5.2E-04	sp286	Bacteria	Firmicutes	Clostridia	Clostridiales	Ruminococcaceae	Pygmaiobacter
sp288_Bacteria_Firmicutes_Negativicutes_Selenomonadales_Veillonellaceae_Megasphaera	genus	0.18	1.4E-03	sp288	Bacteria	Firmicutes	Negativicutes	Selenomonadales	Veillonellaceae	Megasphaera
sp294_Bacteria_Firmicutes_Clostridia_Clostridiales_Ruminococcaceae_Butyricicoccus	genus	0.84	1.5E-03	sp294	Bacteria	Firmicutes	Clostridia	Clostridiales	Ruminococcaceae	Butyricicoccus
sp297_Bacteria_Proteobacteria_Gammaproteobacteria_Enterobacteriales_Enterobacteriaceae_Escherichia/Shigella	genus	0.16	2.4E-03	sp297	Bacteria	Proteobacteria	Gammaproteobacteria	Enterobacteriales	Enterobacteriaceae	Escherichia/Shigella
sp299_Bacteria_Proteobacteria_Gammaproteobacteria_Betaproteobacteriales_Burkholderiaceae_Sutterella	genus	0.38	9.0E-04	sp299	Bacteria	Proteobacteria	Gammaproteobacteria	Betaproteobacteriales	Burkholderiaceae	Sutterella
sp305_Bacteria_Firmicutes_Clostridia_Clostridiales_Ruminococcaceae_Subdoligranulum	genus	0.45	2.0E-03	sp305	Bacteria	Firmicutes	Clostridia	Clostridiales	Ruminococcaceae	Subdoligranulum
sp310_Bacteria_Firmicutes_Clostridia_Clostridiales_Lachnospiraceae_Eisenbergiella	genus	0.56	1.3E-03	sp310	Bacteria	Firmicutes	Clostridia	Clostridiales	Lachnospiraceae	Eisenbergiella
sp311_Bacteria_Firmicutes_Clostridia_Clostridiales_Ruminococcaceae_Anaerotruncus	genus	0.70	9.7E-04	sp311	Bacteria	Firmicutes	Clostridia	Clostridiales	Ruminococcaceae	Anaerotruncus
sp329_Bacteria_Firmicutes_Clostridia_Clostridiales_Lachnospiraceae_Dorea	genus	0.42	1.4E-03	sp329	Bacteria	Firmicutes	Clostridia	Clostridiales	Lachnospiraceae	Dorea
sp338_Bacteria_Actinobacteria_Coriobacteriia_Coriobacteriales_Coriobacteriaceae_Collinsella	genus	0.27	5.0E-04	sp338	Bacteria	Actinobacteria	Coriobacteriia	Coriobacteriales	Coriobacteriaceae	Collinsella
sp341_Bacteria_Firmicutes_Erysipelotrichia_Erysipelotrichales_Erysipelotrichaceae_Turicibacter	genus	0.27	3.5E-04	sp341	Bacteria	Firmicutes	Erysipelotrichia	Erysipelotrichales	Erysipelotrichaceae	Turicibacter

sp343_Bacteria_Firmicutes_Clostridia_Clostridiales_Ruminococcaceae_Intestinimonas	genus	0.79	1.9E-03	sp343	Bacteria	Firmicutes	Clostridia	Clostridiales	Ruminococcaceae	Intestinimonas
sp344_Bacteria_Firmicutes_Bacilli_Lactobacillales_Streptococcaceae_Lactococcus	genus	0.47	4.7E-04	sp344	Bacteria	Firmicutes	Bacilli	Lactobacillales	Streptococcaceae	Lactococcus
sp345_Bacteria_Bacteroidetes_Bacteroidia_Bacteroidales_Marinifilaceae_Odoribacter	genus	0.50	1.1E-03	sp345	Bacteria	Bacteroidetes	Bacteroidia	Bacteroidales	Marinifilaceae	Odoribacter
sp353_Bacteria_Cyanobacteria_Oxyphotobacteria_Chloroplast_NA_NA	genus	0.24	7.0E-04	sp353	Bacteria	Cyanobacteria	Oxyphotobacteria	Chloroplast	NA	NA
sp355_Bacteria_Firmicutes_Clostridia_Clostridiales_Ruminococcaceae_Ruminococcaceae_UCG-002	genus	0.36	2.4E-03	sp355	Bacteria	Firmicutes	Clostridia	Clostridiales	Ruminococcaceae	Ruminococcaceae_UCG-002
sp357_Bacteria_Firmicutes_Clostridia_Clostridiales_Peptostreptococcaceae_Intestinibacter	genus	0.12	7.7E-04	sp357	Bacteria	Firmicutes	Clostridia	Clostridiales	Peptostreptococcaceae	Intestinibacter
sp361_Bacteria_Actinobacteria_Coriobacteriia_Coriobacteriales_Coriobacteriales_Incertae_Sedis_NA	genus	0.41	5.9E-04	sp361	Bacteria	Actinobacteria	Coriobacteriia	Coriobacteriales	Coriobacteriales_Incertae_Sedis	NA
sp371_Bacteria_Firmicutes_Clostridia_Clostridiales_Lachnospiraceae_Lachnospiraceae_NK4A136_group	genus	0.52	2.4E-03	sp371	Bacteria	Firmicutes	Clostridia	Clostridiales	Lachnospiraceae	Lachnospiraceae_NK4A136_group
sp372_Bacteria_Firmicutes_Erysipelotrichia_Erysipelotrichales_Erysipelotrichaceae_Faecalitalea	genus	0.52	1.1E-03	sp372	Bacteria	Firmicutes	Erysipelotrichia	Erysipelotrichales	Erysipelotrichaceae	Faecalitalea
sp374_Bacteria_Firmicutes_Clostridia_Clostridiales_Lachnospiraceae_Marvinbryantia	genus	0.60	1.0E-03	sp374	Bacteria	Firmicutes	Clostridia	Clostridiales	Lachnospiraceae	Marvinbryantia
sp379_Bacteria_Proteobacteria_Gammaproteobacteria_Enterobacteriales_Enterobacteriaceae_Enterobacter	genus	0.10	2.9E-04	sp379	Bacteria	Proteobacteria	Gammaproteobacteria	Enterobacteriales	Enterobacteriaceae	Enterobacter
sp380_Bacteria_Firmicutes_Clostridia_Clostridiales_Peptostreptococcaceae_Romboutsia	genus	0.16	4.0E-04	sp380	Bacteria	Firmicutes	Clostridia	Clostridiales	Peptostreptococcaceae	Romboutsia
sp389_Bacteria_Firmicutes_Clostridia_Clostridiales_Lachnospiraceae_Hungatella	genus	0.48	6.8E-04	sp389	Bacteria	Firmicutes	Clostridia	Clostridiales	Lachnospiraceae	Hungatella
sp403_Bacteria_Firmicutes_Clostridia_Clostridiales_Clostridiaceae_1_Clostridium_sensu_stricto_1	genus	0.30	1.1E-03	sp403	Bacteria	Firmicutes	Clostridia	Clostridiales	Clostridiaceae_1	Clostridium_sensu_stricto_1
sp406_Bacteria_Firmicutes_Clostridia_Clostridiales_Lachnospiraceae_UC5-1-2E3	genus	0.22	2.3E-04	sp406	Bacteria	Firmicutes	Clostridia	Clostridiales	Lachnospiraceae	UC5-1-2E3
sp407_Bacteria_Proteobacteria_Gammaproteobacteria_Pasteurellales_Pasteurellaceae_Haemophilus	genus	0.28	5.6E-04	sp407	Bacteria	Proteobacteria	Gammaproteobacteria	Pasteurellales	Pasteurellaceae	Haemophilus
sp413_Bacteria_Firmicutes_Clostridia_Clostridiales_Ruminococcaceae_Negativibacillus	genus	0.41	9.3E-04	sp413	Bacteria	Firmicutes	Clostridia	Clostridiales	Ruminococcaceae	Negativibacillus
sp440_Bacteria_Firmicutes_Clostridia_Clostridiales_Ruminococcaceae_Ruminiclostridium_6	genus	0.17	7.7E-04	sp440	Bacteria	Firmicutes	Clostridia	Clostridiales	Ruminococcaceae	Ruminiclostridium_6
sp452_Bacteria_Firmicutes_Clostridia_Clostridiales_Family_XIII_NA	genus	0.74	1.1E-03	sp452	Bacteria	Firmicutes	Clostridia	Clostridiales	Family_XIII	NA
sp454_Bacteria_Bacteroidetes_Bacteroidia_Bacteroidales_Barnesiellaceae_Barnesiella	genus	0.28	2.1E-03	sp454	Bacteria	Bacteroidetes	Bacteroidia	Bacteroidales	Barnesiellaceae	Barnesiella
sp468_Bacteria_Firmicutes_Clostridia_Clostridiales_Lachnospiraceae_Tyzzerella_3	genus	0.15	2.9E-04	sp468	Bacteria	Firmicutes	Clostridia	Clostridiales	Lachnospiraceae	Tyzzerella_3
sp471_Bacteria_Firmicutes_Clostridia_Clostridiales_Lachnospiraceae_Agathobacter	genus	0.60	4.8E-03	sp471	Bacteria	Firmicutes	Clostridia	Clostridiales	Lachnospiraceae	Agathobacter
sp497_Bacteria_Actinobacteria_Coriobacteriia_Coriobacteriales_Eggerthellaceae_Eggerthella	genus	0.51	4.1E-04	sp497	Bacteria	Actinobacteria	Coriobacteriia	Coriobacteriales	Eggerthellaceae	Eggerthella
sp507_Bacteria_Firmicutes_Clostridia_Clostridiales_Lachnospiraceae_Shuttleworthia	genus	0.37	8.3E-04	sp507	Bacteria	Firmicutes	Clostridia	Clostridiales	Lachnospiraceae	Shuttleworthia
sp524_Bacteria_Firmicutes_Clostridia_Clostridiales_Christensenellaceae_NA	genus	0.68	6.0E-04	sp524	Bacteria	Firmicutes	Clostridia	Clostridiales	Christensenellaceae	NA
sp531_Bacteria_Firmicutes_Clostridia_Clostridiales_Lachnospiraceae_Lachnospiraceae_FCS020_group	genus	0.58	5.3E-04	sp531	Bacteria	Firmicutes	Clostridia	Clostridiales	Lachnospiraceae	Lachnospiraceae_FCS020_group
sp567_Bacteria_Actinobacteria_Coriobacteriia_Coriobacteriales_Eggerthellaceae_NA	genus	0.18	2.4E-04	sp567	Bacteria	Actinobacteria	Coriobacteriia	Coriobacteriales	Eggerthellaceae	NA
sp576_Bacteria_Firmicutes_Clostridia_Clostridiales_Clostridiales_vadinBB60_group_NA	genus	0.34	8.3E-04	sp576	Bacteria	Firmicutes	Clostridia	Clostridiales	Clostridiales_vadinBB60_group	NA
sp598_Bacteria_Firmicutes_Erysipelotrichia_Erysipelotrichales_Erysipelotrichaceae_NA	genus	0.54	8.5E-04	sp598	Bacteria	Firmicutes	Erysipelotrichia	Erysipelotrichales	Erysipelotrichaceae	NA
sp604_Bacteria_Bacteroidetes_Bacteroidia_Bacteroidales_Barnesiellaceae_NA	genus	0.12	2.4E-04	sp604	Bacteria	Bacteroidetes	Bacteroidia	Bacteroidales	Barnesiellaceae	NA
sp629_Bacteria_Firmicutes_Clostridia_Clostridiales_Ruminococcaceae_Oscillospira	genus	0.26	1.9E-04	sp629	Bacteria	Firmicutes	Clostridia	Clostridiales	Ruminococcaceae	Oscillospira
sp633_Bacteria_Firmicutes_Erysipelotrichia_Erysipelotrichales_Erysipelotrichaceae_Erysipelotrichaceae_UCG-003	genus	0.20	7.0E-04	sp633	Bacteria	Firmicutes	Erysipelotrichia	Erysipelotrichales	Erysipelotrichaceae	Erysipelotrichaceae_UCG-003
sp652_Bacteria_Firmicutes_Clostridia_Clostridiales_Lachnospiraceae_GCA-900066575	genus	0.58	5.0E-04	sp652	Bacteria	Firmicutes	Clostridia	Clostridiales	Lachnospiraceae	GCA-900066575
sp666_Bacteria_Synergistetes_Synergistia_Synergistales_Synergistaceae_Cloacibacillus	genus	0.16	6.0E-04	sp666	Bacteria	Synergistetes	Synergistia	Synergistales	Synergistaceae	Cloacibacillus
sp683_Bacteria_Firmicutes_Clostridia_Clostridiales_Ruminococcaceae_Oscillibacter	genus	0.68	5.1E-04	sp683	Bacteria	Firmicutes	Clostridia	Clostridiales	Ruminococcaceae	Oscillibacter
sp685_Bacteria_Firmicutes_Clostridia_Clostridiales_Ruminococcaceae_Hydrogenoanaerobacterium	genus	0.26	2.5E-04	sp685	Bacteria	Firmicutes	Clostridia	Clostridiales	Ruminococcaceae	Hydrogenoanaerobacterium
sp691_Bacteria_Firmicutes_Erysipelotrichia_Erysipelotrichales_Erysipelotrichaceae_Holdemania	genus	0.44	2.7E-04	sp691	Bacteria	Firmicutes	Erysipelotrichia	Erysipelotrichales	Erysipelotrichaceae	Holdemania
sp731_Bacteria_Firmicutes_Clostridia_Clostridiales_Lachnospiraceae_Fusicatenibacter	genus	0.28	1.2E-03	sp731	Bacteria	Firmicutes	Clostridia	Clostridiales	Lachnospiraceae	Fusicatenibacter
sp733_Bacteria_Actinobacteria_Coriobacteriia_Coriobacteriales_Eggerthellaceae_Adlercreutzia	genus	0.52	3.7E-04	sp733	Bacteria	Actinobacteria	Coriobacteriia	Coriobacteriales	Eggerthellaceae	Adlercreutzia
sp749_Bacteria_Firmicutes_Clostridia_Clostridiales_Ruminococcaceae_UBA1819	genus	0.32	5.2E-04	sp749	Bacteria	Firmicutes	Clostridia	Clostridiales	Ruminococcaceae	UBA1819
sp753_Bacteria_Firmicutes_Erysipelotrichia_Erysipelotrichales_Erysipelotrichaceae_Coprobacillus	genus	0.22	1.7E-04	sp753	Bacteria	Firmicutes	Erysipelotrichia	Erysipelotrichales	Erysipelotrichaceae	Coprobacillus
sp772_Bacteria_Firmicutes_Erysipelotrichia_Erysipelotrichales_Erysipelotrichaceae_Dielma	genus	0.39	1.8E-04	sp772	Bacteria	Firmicutes	Erysipelotrichia	Erysipelotrichales	Erysipelotrichaceae	Dielma
sp789_Bacteria_Firmicutes_Clostridia_Clostridiales_Ruminococcaceae_Ruminiclostridium	genus	0.11	3.2E-04	sp789	Bacteria	Firmicutes	Clostridia	Clostridiales	Ruminococcaceae	Ruminiclostridium
sp796_Bacteria_Firmicutes_Clostridia_Clostridiales_Ruminococcaceae_Caproiciproducens	genus	0.44	2.2E-04	sp796	Bacteria	Firmicutes	Clostridia	Clostridiales	Ruminococcaceae	Caproiciproducens
sp800_Bacteria_Firmicutes_Clostridia_Clostridiales_Lachnospiraceae_GCA-900066755	genus	0.32	1.2E-04	sp800	Bacteria	Firmicutes	Clostridia	Clostridiales	Lachnospiraceae	GCA-900066755
sp809_Bacteria_Firmicutes_Clostridia_Clostridiales_Ruminococcaceae_Ruminococcaceae_UCG-004	genus	0.21	3.9E-04	sp809	Bacteria	Firmicutes	Clostridia	Clostridiales	Ruminococcaceae	Ruminococcaceae_UCG-004
sp817_Bacteria_Actinobacteria_Coriobacteriia_Coriobacteriales_Eggerthellaceae_Senegalimassilia	genus	0.16	2.1E-04	sp817	Bacteria	Actinobacteria	Coriobacteriia	Coriobacteriales	Eggerthellaceae	Senegalimassilia
sp818_Bacteria_Firmicutes_Clostridia_Clostridiales_Ruminococcaceae_Phocea	genus	0.42	1.1E-04	sp818	Bacteria	Firmicutes	Clostridia	Clostridiales	Ruminococcaceae	Phocea
sp826_Bacteria_Firmicutes_Negativicutes_Selenomonadales_Veillonellaceae_Veillonella	genus	0.24	3.9E-04	sp826	Bacteria	Firmicutes	Negativicutes	Selenomonadales	Veillonellaceae	Veillonella
sp836_Bacteria_Firmicutes_Clostridia_Clostridiales_Lachnospiraceae_Anaerosporobacter	genus	0.11	1.6E-04	sp836	Bacteria	Firmicutes	Clostridia	Clostridiales	Lachnospiraceae	Anaerosporobacter
sp858_Bacteria_Firmicutes_Clostridia_Clostridiales_Ruminococcaceae_GCA-900066225	genus	0.59	5.8E-04	sp858	Bacteria	Firmicutes	Clostridia	Clostridiales	Ruminococcaceae	GCA-900066225
sp908_Bacteria_Firmicutes_Erysipelotrichia_Erysipelotrichales_Erysipelotrichaceae_Solobacterium	genus	0.13	1.1E-04	sp908	Bacteria	Firmicutes	Erysipelotrichia	Erysipelotrichales	Erysipelotrichaceae	Solobacterium
sp959_Bacteria_Bacteroidetes_Bacteroidia_Bacteroidales_Prevotellaceae_Prevotella_7	genus	0.15	1.3E-04	sp959	Bacteria	Bacteroidetes	Bacteroidia	Bacteroidales	Prevotellaceae	Prevotella_7
sp963_Bacteria_Firmicutes_Clostridia_Clostridiales_Ruminococcaceae_Candidatus_Soleaferrea	genus	0.67	2.1E-04	sp963	Bacteria	Firmicutes	Clostridia	Clostridiales	Ruminococcaceae	Candidatus_Soleaferrea
sp978_Bacteria_Tenericutes_Mollicutes_Mollicutes_RF39_NA_NA	genus	0.21	1.6E-03	sp978	Bacteria	Tenericutes	Mollicutes	Mollicutes_RF39	NA	NA
sp982_Bacteria_Actinobacteria_Coriobacteriia_Coriobacteriales_Eggerthellaceae_Slackia	genus	0.27	1.5E-04	sp982	Bacteria	Actinobacteria	Coriobacteriia	Coriobacteriales	Eggerthellaceae	Slackia
sp1020_Bacteria_Firmicutes_Erysipelotrichia_Erysipelotrichales_Erysipelotrichaceae_Merdibacter	genus	0.23	2.3E-04	sp1020	Bacteria	Firmicutes	Erysipelotrichia	Erysipelotrichales	Erysipelotrichaceae	Merdibacter
sp1048_Bacteria_Bacteroidetes_Bacteroidia_Bacteroidales_Marinifilaceae_Butyricimonas	genus	0.33	4.5E-04	sp1048	Bacteria	Bacteroidetes	Bacteroidia	Bacteroidales	Marinifilaceae	Butyricimonas
sp1132_Bacteria_Firmicutes_Clostridia_Clostridiales_Ruminococcaceae_Ruminiclostridium_1	genus	0.21	2.5E-04	sp1132	Bacteria	Firmicutes	Clostridia	Clostridiales	Ruminococcaceae	Ruminiclostridium_1
sp1138_Bacteria_Firmicutes_Negativicutes_Selenomonadales_Veillonellaceae_Allisonella	genus	0.11	1.2E-04	sp1138	Bacteria	Firmicutes	Negativicutes	Selenomonadales	Veillonellaceae	Allisonella
sp1198_Bacteria_Firmicutes_Clostridia_Clostridiales_Ruminococcaceae_Ruminococcaceae_UCG-010	genus	0.48	1.1E-03	sp1198	Bacteria	Firmicutes	Clostridia	Clostridiales	Ruminococcaceae	Ruminococcaceae_UCG-010
sp1213_Bacteria_Firmicutes_Clostridia_Clostridiales_Peptococcaceae_Peptococcus	genus	0.21	1.7E-04	sp1213	Bacteria	Firmicutes	Clostridia	Clostridiales	Peptococcaceae	Peptococcus
sp1254_Bacteria_Firmicutes_Clostridia_Clostridiales_Lachnospiraceae_Tyzzerella	genus	0.15	2.0E-04	sp1254	Bacteria	Firmicutes	Clostridia	Clostridiales	Lachnospiraceae	Tyzzerella
sp1261_Bacteria_Proteobacteria_Gammaproteobacteria_Betaproteobacteriales_Burkholderiaceae_Oxalobacter	genus	0.24	1.0E-04	sp1261	Bacteria	Proteobacteria	Gammaproteobacteria	Betaproteobacteriales	Burkholderiaceae	Oxalobacter
sp1325_Bacteria_Proteobacteria_Gammaproteobacteria_Enterobacteriales_Enterobacteriaceae_NA	genus	0.17	1.6E-04	sp1325	Bacteria	Proteobacteria	Gammaproteobacteria	Enterobacteriales	Enterobacteriaceae	NA
sp1361_Bacteria_Firmicutes_Clostridia_Clostridiales_Peptococcaceae_NA	genus	0.44	2.3E-04	sp1361	Bacteria	Firmicutes	Clostridia	Clostridiales	Peptococcaceae	NA
sp1399_Bacteria_Firmicutes_Clostridia_Clostridiales_Ruminococcaceae_Ruminococcaceae_UCG-013	genus	0.43	4.4E-04	sp1399	Bacteria	Firmicutes	Clostridia	Clostridiales	Ruminococcaceae	Ruminococcaceae_UCG-013
sp1434_Bacteria_Proteobacteria_Alphaproteobacteria_Rhodospirillales_NA_NA	genus	0.16	2.4E-04	sp1434	Bacteria	Proteobacteria	Alphaproteobacteria	Rhodospirillales	NA	NA
sp1500_Bacteria_Actinobacteria_Coriobacteriia_Coriobacteriales_Eggerthellaceae_Gordonibacter	genus	0.26	1.1E-04	sp1500	Bacteria	Actinobacteria	Coriobacteriia	Coriobacteriales	Eggerthellaceae	Gordonibacter
sp1737_Bacteria_Bacteroidetes_Bacteroidia_Bacteroidales_Prevotellaceae_Prevotella	genus	0.13	1.0E-04	sp1737	Bacteria	Bacteroidetes	Bacteroidia	Bacteroidales	Prevotellaceae	Prevotella
sp1802_Bacteria_Firmicutes_Clostridia_Clostridiales_Defluviitaleaceae_Defluviitaleaceae_UCG-011	genus	0.47	2.6E-04	sp1802	Bacteria	Firmicutes	Clostridia	Clostridiales	Defluviitaleaceae	Defluviitaleaceae_UCG-011
sp2043_Bacteria_Firmicutes_Clostridia_Clostridiales_NA_NA	genus	0.25	1.5E-04	sp2043	Bacteria	Firmicutes	Clostridia	Clostridiales	NA	NA
sp2094_Bacteria_Proteobacteria_Deltaproteobacteria_Desulfovibrionales_Desulfovibrionaceae_Bilophila	genus	0.13	1.2E-04	sp2094	Bacteria	Proteobacteria	Deltaproteobacteria	Desulfovibrionales	Desulfovibrionaceae	Bilophila

Supplementary Table 2: Brown taxa module membership and taxa significance for clinical traits correlated with the covariate adjusted brown module (GAD7, PHQ9, DARS)**.** Taxa significance is measured by the correlation (Pearson) between each taxon and clinical trait. Brown module membership is the correlation between the individual taxon and the brown. module eigentaxon. High module membership is a proxy measurement for highly connected taxa and therefore module hub taxa are defined by high module membership.

	Brown Module Membership	GAD7				PHQ9				DARS		
		r	p	p.BH		r	p	p.BH		r	p	p.BH
**sp1198_Ruminococcaceae_UCG-010**	0.81	-0.3	8.13E-05	0.005	**	-0.23	3.68E-03	0.053	.	0.12	1.17E-01	0.366
**sp161_Christensenellaceae_R-7_group**	0.72	-0.21	8.16E-03	0.057	.	-0.17	2.78E-02	0.216		0.15	5.63E-02	0.292
**sp576_Clostridiales_vadinBB60_group_NA**	0.69	-0.25	1.54E-03	0.027	*	-0.11	1.64E-01	0.485		0.14	7.68E-02	0.292
sp105_Ruminococcaceae_UCG-005	0.68	-0.2	1.02E-02	0.057	.	-0.07	3.47E-01	0.58		0.05	5.08E-01	0.813
sp1132_Ruminiclostridium_1	0.64	-0.24	1.90E-03	0.027	*	-0.19	1.32E-02	0.158		0.12	1.36E-01	0.402
sp280_Ruminococcaceae_NK4A214_group	0.64	-0.3	1.30E-04	0.005	**	-0.19	1.67E-02	0.172		0.17	3.10E-02	0.292
sp107_Ruminococcaceae_UCG-002	0.63	-0.16	3.55E-02	0.111		-0.11	1.71E-01	0.485		0.02	8.28E-01	0.885
sp1361_Peptococcaceae_NA	0.63	-0.25	1.19E-03	0.027	*	-0.27	4.63E-04	0.017	*	0.16	4.19E-02	0.292
sp240_Christensenellaceae_R-7_group	0.6	-0.17	2.98E-02	0.103		-0.09	2.36E-01	0.548		0.11	1.67E-01	0.43
sp155_Christensenellaceae_R-7_group	0.6	-0.2	1.02E-02	0.057	.	-0.15	6.47E-02	0.262		0.09	2.68E-01	0.568
sp224_Ruminococcaceae_UCG-005	0.6	-0.21	7.03E-03	0.057	.	-0.16	4.24E-02	0.235		0.1	1.97E-01	0.443
sp79_Ruminococcaceae_UCG-002	0.59	-0.15	5.45E-02	0.157		-0.01	8.59E-01	0.884		0.05	5.20E-01	0.814
sp2043_NA_NA	0.54	-0.2	1.11E-02	0.057	.	-0.16	4.60E-02	0.237		0.2	8.83E-03	0.194
sp156_Ruminococcaceae_NK4A214_group	0.52	-0.21	7.76E-03	0.057	.	-0.24	2.04E-03	0.037	*	0.2	1.08E-02	0.194
sp268_Desulfovibrio	0.51	0.04	6.44E-01	0.773		0.05	5.06E-01	0.637		-0.02	7.73E-01	0.864
sp355_Ruminococcaceae_UCG-002	0.51	-0.17	2.99E-02	0.103		-0.17	3.00E-02	0.216		0.14	6.86E-02	0.292
sp345_Odoribacter	0.5	-0.1	2.16E-01	0.409		-0.05	5.13E-01	0.637		0.07	3.71E-01	0.652
sp978_NA_NA	0.47	-0.12	1.19E-01	0.275		-0.08	3.11E-01	0.58		0.05	5.59E-01	0.834
sp1399_Ruminococcaceae_UCG-013	0.46	-0.13	1.07E-01	0.262		-0.07	3.74E-01	0.58		0.04	6.03E-01	0.834
sp193_Ruminococcaceae_UCG-014	0.46	-0.2	9.98E-03	0.057	.	-0.14	7.56E-02	0.287		-0.02	7.68E-01	0.864
sp666_Cloacibacillus	0.45	-0.09	2.56E-01	0.46		-0.05	5.13E-01	0.637		0.16	4.64E-02	0.292
sp208_Ruminococcaceae_UCG-002	0.44	-0.12	1.33E-01	0.291		-0.02	8.22E-01	0.869		0.13	9.31E-02	0.319
sp201_Christensenellaceae_R-7_group	0.43	-0.12	1.32E-01	0.291		-0.1	2.03E-01	0.517		0.03	7.32E-01	0.864
sp148_Alistipes	0.38	-0.19	1.45E-02	0.065	.	-0.16	3.79E-02	0.228		0.14	7.05E-02	0.292
sp1048_Butyricimonas	0.38	-0.08	3.13E-01	0.513		-0.07	3.47E-01	0.58		0.01	8.56E-01	0.893
sp1261_Oxalobacter	0.38	-0.05	5.32E-01	0.672		-0.07	3.87E-01	0.581		0.02	8.36E-01	0.885
sp94_Ruminiclostridium_6	0.37	-0.19	1.39E-02	0.065	.	-0.1	2.10E-01	0.517		0.04	6.53E-01	0.855
sp226_Family_XIII_AD3011_group	0.37	-0.11	1.57E-01	0.322		-0.06	4.18E-01	0.614		0.13	8.81E-02	0.317
sp1802_Defluviitaleaceae_UCG-011	0.35	-0.19	1.67E-02	0.071	.	-0.26	8.61E-04	0.021	*	0.12	1.40E-01	0.402
sp440_Ruminiclostridium_6	0.34	-0.09	2.41E-01	0.445		0.02	8.33E-01	0.869		0.14	7.35E-02	0.292
sp454_Barnesiella	0.34	-0.07	4.08E-01	0.576		0.05	4.87E-01	0.637		0.03	7.44E-01	0.864
sp97_Bacteroides	0.31	-0.19	1.76E-02	0.071	.	-0.07	3.79E-01	0.58		0.08	3.39E-01	0.626
sp30_Alistipes	0.3	-0.13	1.02E-01	0.262		-0.1	1.97E-01	0.517		0.04	6.01E-01	0.834
sp173_Ruminococcus_1	0.29	-0.07	3.91E-01	0.57		-0.09	2.59E-01	0.58		0.06	4.19E-01	0.702
sp255_Ruminococcaceae_NA	0.29	-0.13	1.09E-01	0.262		-0.05	5.43E-01	0.641		0.07	3.71E-01	0.652
sp361_Coriobacteriales_Incertae_Sedis_NA	0.28	-0.14	8.41E-02	0.224		-0.12	1.35E-01	0.44		0.07	4.09E-01	0.7
sp27_Alistipes	0.28	-0.14	8.22E-02	0.224		-0.11	1.45E-01	0.454		0.1	1.95E-01	0.443
sp219_Parabacteroides	0.26	-0.1	2.00E-01	0.399		-0.08	2.86E-01	0.58		0.15	5.30E-02	0.292
sp51_Roseburia	0.25	-0.15	5.42E-02	0.157		-0.15	6.15E-02	0.262		0.01	8.82E-01	0.907
sp262_Family_XIII_UCG-001	0.24	-0.05	5.13E-01	0.666		-0.11	1.75E-01	0.485		-0.02	7.78E-01	0.864
sp817_Senegalimassilia	0.24	-0.01	8.72E-01	0.924		0.02	8.28E-01	0.869		0	9.91E-01	0.991
sp128_Alistipes	0.23	-0.01	8.51E-01	0.915		0.1	2.16E-01	0.517		0.11	1.82E-01	0.437
sp82_Ruminococcaceae_NA	0.23	0.07	3.51E-01	0.562		0.07	3.60E-01	0.58		-0.01	9.08E-01	0.921
sp139_Lachnospira	0.21	-0.09	2.64E-01	0.464		-0.06	4.73E-01	0.637		0.03	7.29E-01	0.864
sp23_Parabacteroides	0.2	-0.11	1.54E-01	0.322		-0.12	1.14E-01	0.393		0.02	7.56E-01	0.864
sp567_Eggerthellaceae_NA	0.19	-0.02	8.09E-01	0.911		-0.04	6.10E-01	0.709		-0.13	1.06E-01	0.348
sp185_Alistipes	0.19	0	9.83E-01	0.985		0.06	4.27E-01	0.615		-0.08	3.12E-01	0.592
sp1434_NA_NA	0.18	-0.05	5.18E-01	0.666		-0.07	3.64E-01	0.58		0.11	1.49E-01	0.402
sp175_Roseburia	0.16	0.06	4.31E-01	0.596		0	9.61E-01	0.961		-0.04	6.33E-01	0.855
sp191_Prevotella_9	0.16	0.1	2.05E-01	0.399		0.14	6.56E-02	0.262		-0.15	5.95E-02	0.292
sp7_Bacteroides	0.16	-0.22	4.34E-03	0.052	.	-0.13	1.05E-01	0.377		0.03	7.17E-01	0.864
sp71_Roseburia	0.15	-0.21	8.20E-03	0.057	.	-0.16	3.54E-02	0.228		0.06	4.41E-01	0.721
sp17_Bacteroides	0.15	-0.08	3.13E-01	0.513		-0.01	9.38E-01	0.951		0.17	2.58E-02	0.292
sp103_Alistipes	0.14	0.07	3.86E-01	0.57		0.02	7.61E-01	0.856		0.02	7.92E-01	0.864
sp77_Ruminococcaceae_NA	0.14	-0.03	7.15E-01	0.818		-0.02	8.30E-01	0.869		0.09	2.30E-01	0.502
sp171_Lachnospira	0.14	-0.18	2.44E-02	0.092	.	-0.15	6.18E-02	0.262		0.02	7.91E-01	0.864
sp120_Roseburia	0.13	-0.07	3.65E-01	0.57		-0.08	3.24E-01	0.58		0.15	5.46E-02	0.292
sp58_Bacteroides	0.12	-0.04	5.88E-01	0.718		0.05	5.42E-01	0.641		0.03	6.92E-01	0.864
sp278_Mogibacterium	0.12	0.07	3.96E-01	0.57		0.08	3.05E-01	0.58		-0.08	2.86E-01	0.578
sp221_Ruminococcaceae_UCG-002	0.11	0.02	8.36E-01	0.915		0.02	7.87E-01	0.869		-0.04	5.96E-01	0.834
sp198_Muribaculaceae_NA	0.11	0.03	7.16E-01	0.818		0.08	3.34E-01	0.58		-0.08	3.02E-01	0.587
sp1213_Peptococcus	0.11	-0.05	5.12E-01	0.666		-0.08	3.08E-01	0.58		0.04	6.45E-01	0.855
sp76_Catenibacterium	0.1	-0.07	3.85E-01	0.57		-0.08	3.26E-01	0.58		0.05	5.46E-01	0.834
sp151_Catenibacterium	0.1	-0.04	5.78E-01	0.717		-0.07	3.63E-01	0.58		-0.04	5.75E-01	0.834
sp279_Paraprevotella	0.09	0	9.65E-01	0.985		0.05	5.06E-01	0.637		-0.11	1.76E-01	0.437
sp108_Holdemanella	0.07	-0.03	6.82E-01	0.805		0.03	6.83E-01	0.78		-0.16	4.33E-02	0.292
sp158_Lachnospiraceae_NA	0.03	0	9.85E-01	0.985		0.05	4.93E-01	0.637		-0.11	1.51E-01	0.402
sp959_Prevotella_7	0.03	0.17	3.36E-02	0.11		0.28	3.70E-04	0.017	*	-0.18	2.12E-02	0.292
sp64_Erysipelotrichaceae_UCG-003	0.03	-0.08	3.04E-01	0.513		0.05	5.22E-01	0.637		-0.08	2.89E-01	0.578
sp126_Ruminococcaceae_NA	0.02	-0.01	8.51E-01	0.915		0.06	4.47E-01	0.632		-0.14	7.70E-02	0.292
sp299_Sutterella	0	0	9.83E-01	0.985		0.17	2.67E-02	0.216		-0.24	2.11E-03	0.102
sp982_Slackia	0	0.06	4.63E-01	0.629		0.09	2.70E-01	0.58		-0.23	2.83E-03	0.102

**Supplementary Table S3:** Module membership of unadjusted brown module taxa, generated from	
Taxa	Brown Module Membership
**sp1198_ruminococcaceae_ruminococcaceae_ucg_010**	**0.80**
**sp161_christensenellaceae_christensenellaceae_r_7_group**	**0.75**
**sp576_clostridiales_vadin_bb60_group_na**	**0.70**
sp1361_peptococcaceae_na	0.69
sp224_ruminococcaceae_ruminococcaceae_ucg_005	0.68
sp280_ruminococcaceae_ruminococcaceae_nk4a214_group	0.67
sp105_ruminococcaceae_ruminococcaceae_ucg_005	0.66
sp1132_ruminococcaceae_ruminiclostridium_1	0.63
sp156_ruminococcaceae_ruminococcaceae_nk4a214_group	0.61
sp107_ruminococcaceae_ruminococcaceae_ucg_002	0.58
sp240_christensenellaceae_christensenellaceae_r_7_group	0.57
sp2043_na_na	0.55
sp79_ruminococcaceae_ruminococcaceae_ucg_002	0.53
sp193_ruminococcaceae_ruminococcaceae_ucg_014	0.53
sp155_christensenellaceae_christensenellaceae_r_7_group	0.53
sp685_ruminococcaceae_hydrogenoanaerobacterium	0.52
sp1399_ruminococcaceae_ruminococcaceae_ucg_013	0.51
sp343_ruminococcaceae_intestinimonas	0.49
sp858_ruminococcaceae_gca_900066225	0.49
sp201_christensenellaceae_christensenellaceae_r_7_group	0.48
sp1802_defluviitaleaceae_defluviitaleaceae_ucg_011	0.48
sp355_ruminococcaceae_ruminococcaceae_ucg_002	0.47
sp148_rikenellaceae_alistipes	0.46
sp666_synergistaceae_cloacibacillus	0.45
sp978_na_na	0.45
sp226_family_xiii_family_xiii_ad3011_group	0.44
sp94_ruminococcaceae_ruminiclostridium_6	0.44
sp255_ruminococcaceae_na	0.43
sp345_marinifilaceae_odoribacter	0.42
sp524_christensenellaceae_na	0.42
sp1261_burkholderiaceae_oxalobacter	0.39
sp268_desulfovibrionaceae_desulfovibrio	0.39
sp208_ruminococcaceae_ruminococcaceae_ucg_002	0.39
sp1048_marinifilaceae_butyricimonas	0.39
sp97_bacteroidaceae_bacteroides	0.36
sp311_ruminococcaceae_anaerotruncus	0.36
sp683_ruminococcaceae_oscillibacter	0.35
sp262_family_xiii_family_xiii_ucg_001	0.34
sp749_ruminococcaceae_uba1819	0.33
sp361_coriobacteriales_incertae_sedis_na	0.33
sp223_ruminococcaceae_anaerofilum	0.33
sp27_rikenellaceae_alistipes	0.32

sp173_ruminococcaceae_ruminococcus_1	0.32
sp30_rikenellaceae_alistipes	0.32
sp454_barnesiellaceae_barnesiella	0.31
sp139_lachnospiraceae_lachnospira	0.30
sp440_ruminococcaceae_ruminiclostridium_6	0.30
sp71_lachnospiraceae_roseburia	0.29
sp219_tannerellaceae_parabacteroides	0.27
sp1434_na_na	0.26
sp17_bacteroidaceae_bacteroides	0.25
sp128_rikenellaceae_alistipes	0.23
sp199_ruminococcaceae_ruminiclostridium_9	0.21
sp171_lachnospiraceae_lachnospira	0.20
sp371_lachnospiraceae_lachnospiraceae_nk4a136_group	0.19
sp51_lachnospiraceae_roseburia	0.18
sp7_bacteroidaceae_bacteroides	0.17
sp23_tannerellaceae_parabacteroides	0.17
sp185_rikenellaceae_alistipes	0.16
sp170_acidaminococcaceae_phascolarctobacterium	0.14
sp652_lachnospiraceae_gca_900066575	0.12
sp836_lachnospiraceae_anaerosporobacter	0.11
sp18_akkermansiaceae_akkermansia	0.10
sp733_eggerthellaceae_adlercreutzia	0.10
sp77_ruminococcaceae_na	0.10
sp120_lachnospiraceae_roseburia	0.09
sp103_rikenellaceae_alistipes	0.09
sp47_lachnospiraceae_lachnospiraceae_nk4a136_group	0.08
sp64_erysipelotrichaceae_erysipelotrichaceae_ucg_003	0.08
sp175_lachnospiraceae_roseburia	0.08
sp206_ruminococcaceae_ruminococcus_2	0.07
sp58_bacteroidaceae_bacteroides	0.07
sp221_ruminococcaceae_ruminococcaceae_ucg_002	0.07
sp65_bacteroidaceae_bacteroides	0.04
sp93_lachnospiraceae_na	0.02
sp39_ruminococcaceae_ruminococcus_2	0.02
sp3_bacteroidaceae_bacteroides	-0.01
sp69_bacteroidaceae_bacteroides	-0.02
sp116_ruminococcaceae_negativibacillus	-0.04
sp14_bacteroidaceae_bacteroides	-0.05
sp9_bacteroidaceae_bacteroides	-0.05

Supplementary Table 4: Taxa significantly associated with a clinical trait before correction. Association between CLR transformed taxon abundance and clinical scores calculated ALDEx2 glm function.

clinical trait	taxa	β	X Intercept	p	p.BH
GAD7	sp7_Bacteroides	-0.22	3.4	0.013	0.898
GAD7	sp28_Lachnospiraceae_NA	0.28	-0.4	0.001	0.354
GAD7	sp45_Roseburia	0.17	-0.9	0.04	0.996
GAD7	sp71_Roseburia	-0.2	3.5	0.01	0.790
GAD7	sp94_Ruminiclostridium_6	-0.17	1.3	0.025	0.951
GAD7	sp109_Faecalitalea	0.17	-1.9	0.035	0.963
GAD7	sp122_Lachnoclostridium	0.16	-1.4	0.034	0.978
GAD7	sp152_Blautia	0.19	-3.4	0.021	0.769
GAD7	sp148_Alistipes	-0.15	5.0	0.029	0.985
GAD7	sp161_Christensenellaceae_R-7_group	-0.16	3.6	0.031	0.972
GAD7	sp193_Ruminococcaceae_UCG-014	-0.19	2.2	0.02	0.917
GAD7	sp195_Lachnoclostridium	0.1	5.8	0.008	0.953
GAD7	sp224_Ruminococcaceae_UCG-005	-0.15	3.9	0.015	0.879
GAD7	sp244_Bifidobacterium	0.22	-0.9	0.003	0.556
GAD7	sp280_Ruminococcaceae_NK4A214_group	-0.22	1.4	0.004	0.503
GAD7	sp343_Intestinimonas	-0.12	3.8	0.039	0.975
GAD7	sp372_Faecalitalea	0.19	-1.3	0.01	0.777
GAD7	sp380_Romboutsia	0.17	-3.8	0.015	0.695
GAD7	sp524_Christensenellaceae_NA	-0.18	2.5	0.001	0.243
GAD7	sp1198_Ruminococcaceae_UCG-010	-0.22	2.0	<0.001	0.195
GAD7	sp1361_Peptococcaceae_NA	-0.14	0.2	0.027	0.777
PHQ-9	sp19_Blautia	0.12	3.6	0.039	0.998
PHQ-9	sp28_Lachnospiraceae_NA	0.17	0.0	0.03	0.991
PHQ-9	sp71_Roseburia	-0.15	3.4	0.02	0.950
PHQ-9	sp109_Faecalitalea	0.15	-2.3	0.026	0.907
PHQ-9	sp129_Lachnoclostridium	0.15	-1.1	0.013	0.864
PHQ-9	sp152_Blautia	0.13	-3.2	0.045	0.944
PHQ-9	sp195_Lachnoclostridium	0.08	5.8	0.014	1.000
PHQ-9	sp224_Ruminococcaceae_UCG-005	-0.1	3.8	0.046	0.993
PHQ-9	sp244_Bifidobacterium	0.15	-0.7	0.017	0.948
PHQ-9	sp280_Ruminococcaceae_NK4A214_group	-0.15	1.1	0.029	0.901
PHQ-9	sp288_Megasphaera	0.15	-3.6	0.022	0.815
PHQ-9	sp299_Sutterella	0.12	-1.9	0.048	0.966
PHQ-9	sp343_Intestinimonas	-0.13	4.2	0.007	0.759
PHQ-9	sp372_Faecalitalea	0.13	-1.2	0.04	0.973
PHQ-9	sp380_Romboutsia	0.12	-3.7	0.039	0.874
PHQ-9	sp524_Christensenellaceae_NA	-0.15	2.6	0.002	0.294
PHQ-9	sp1198_Ruminococcaceae_UCG-010	-0.14	1.7	0.013	0.792
PHQ-9	sp1361_Peptococcaceae_NA	-0.13	0.5	0.008	0.673
PHQ-9	sp1802_Defluviitaleaceae_UCG-011	-0.12	0.6	0.019	0.769
DARS	sp55_Bifidobacterium	-0.09	3.5	0.015	0.873
DARS	sp104_Streptococcus	0.08	-4.2	0.022	0.850
DARS	sp299_Sutterella	-0.07	2.6	0.023	0.832
