# Supplementary material for: Leveraging the microbiome to understand clinical heterogeneity in depression: findings from the T-RAD study
Source: Transl Psychiatry. 2023 Apr 28;13:139. doi: 10.1038/s41398-023-02416-3 (PMC10147668; doi:10.1038/s41398-023-02416-3)
Supplement: Supplementary file 2 — Supplemental Figures [file 41398_2023_2416_MOESM2_ESM.pdf]

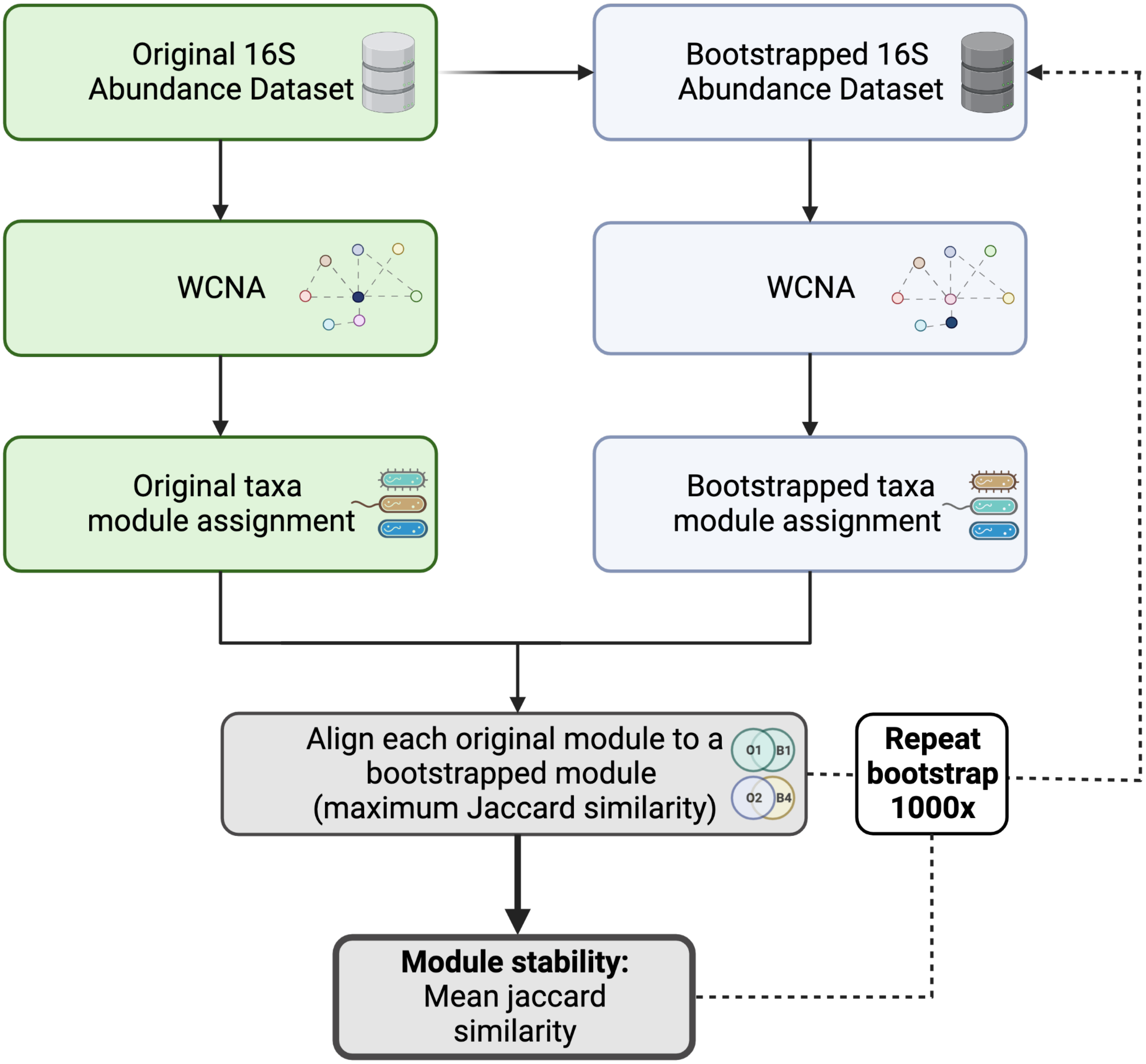

Supplementary Figure S1: Schematic summarizing WCNA module stability algorithm. Module stability was determined by calculating the mean Jaccard similarity between taxa in the original module and 1000 bootstrapped modules. The bootstrapped module in each iteration with highest Jaccard similarity with the original module was used to calculate the mean. Higher mean Jaccard similarity indicates the module has more stable taxa membership.

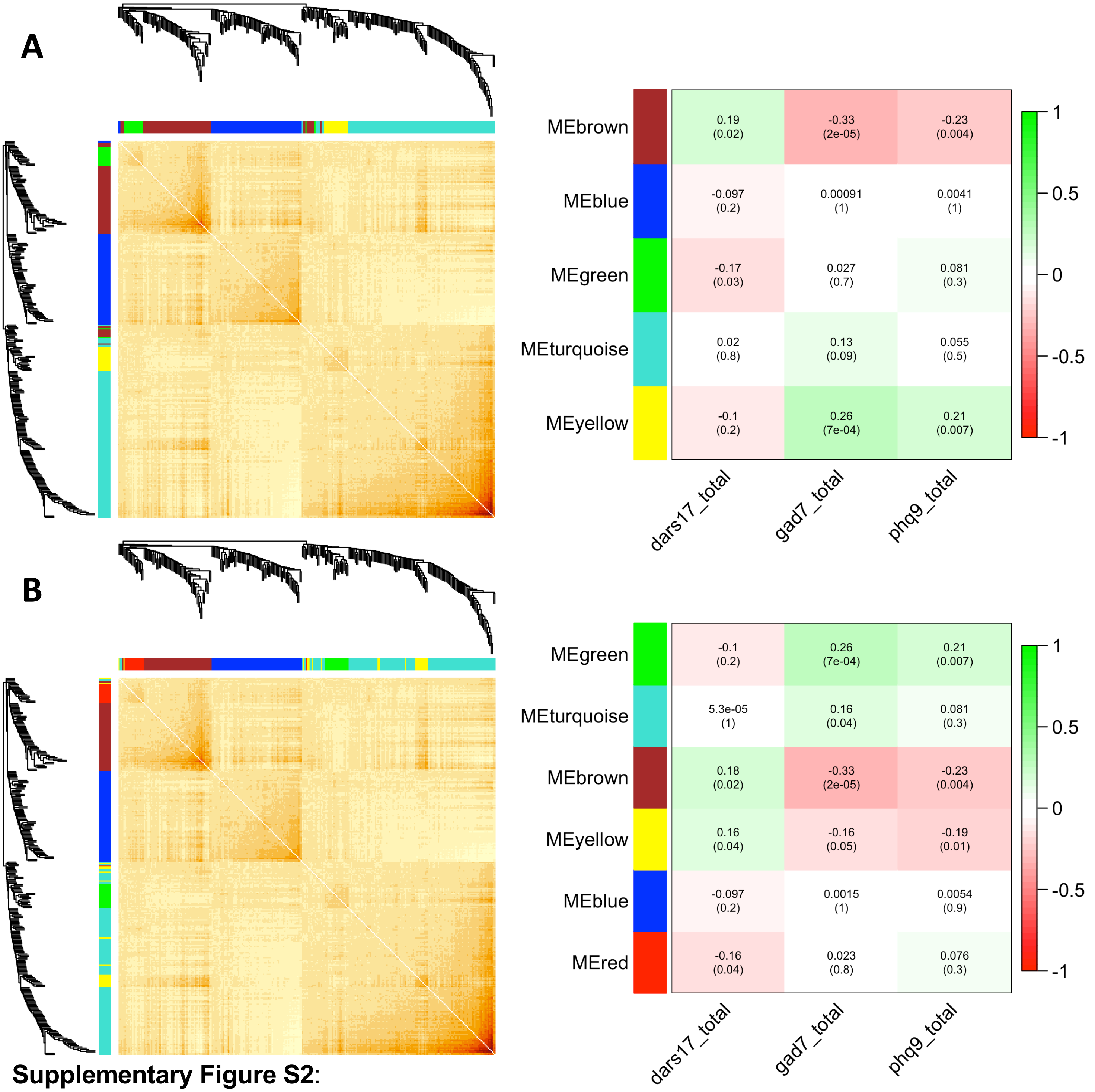

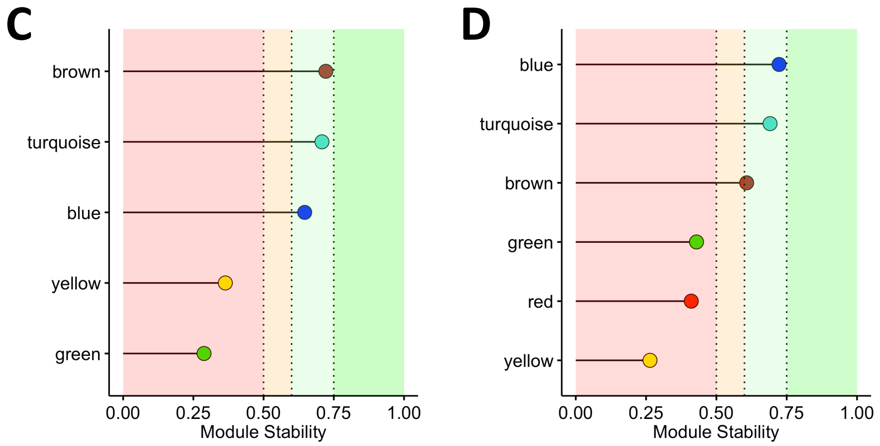

Supplementary Figure S2: Alternative minimum module size parameters for covariate corrected WCNA had unstable modules. **A)** Minimum module size 15 network and module-trait correlations. **B)** Minimum module size 10 network and module-trait correlations **C)** Module stability of network with minimum module size 15. Yellow and green modules are unstable. **D)** Module stability of network with minimum module size 10. Green, red, and yellow modules are unstable.

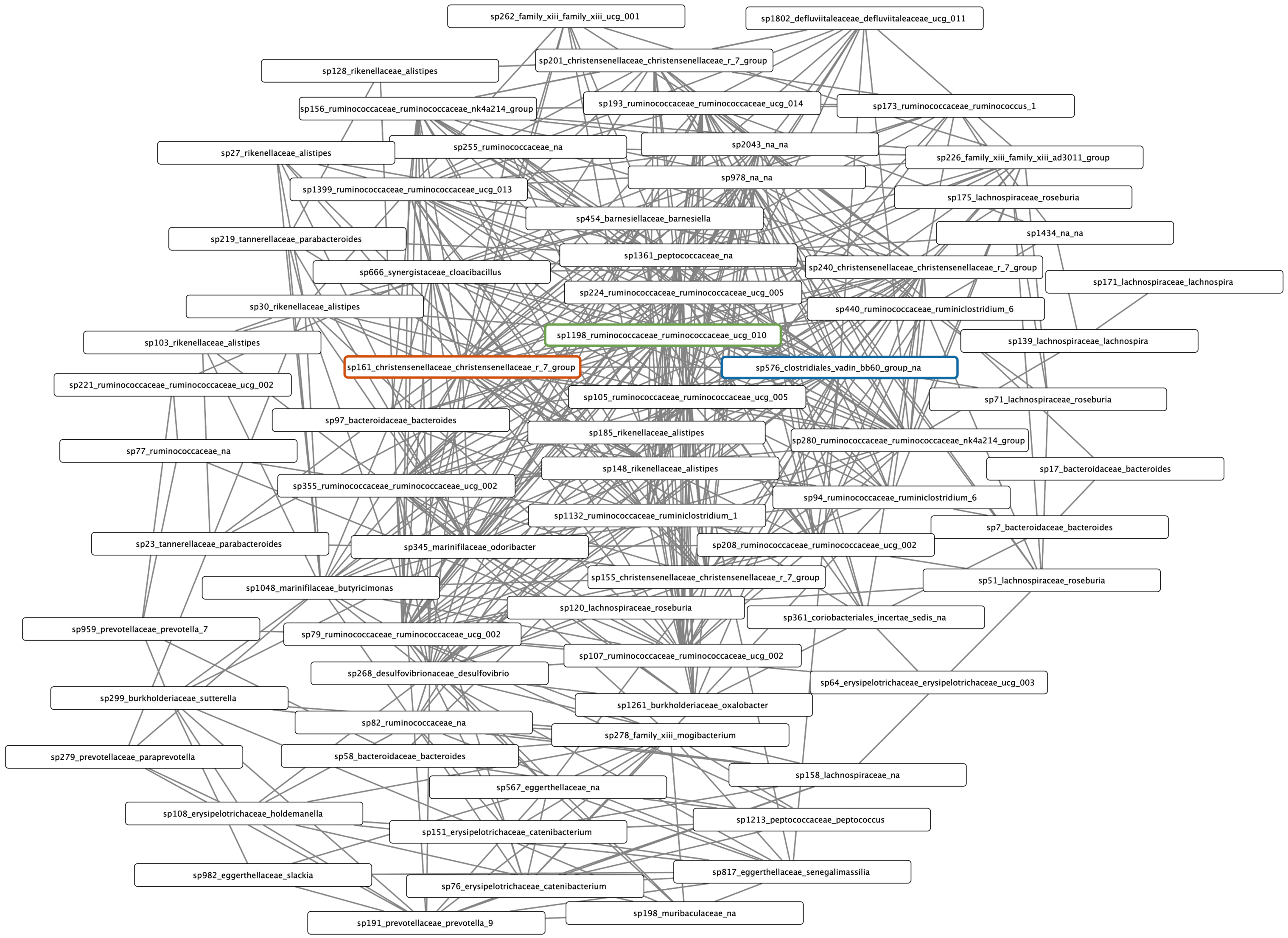

Supplementary Figure S3: Visualization of the covariate corrected brown gut microbial network as a correlation network pruned to connections with Pearson R > 0.2.

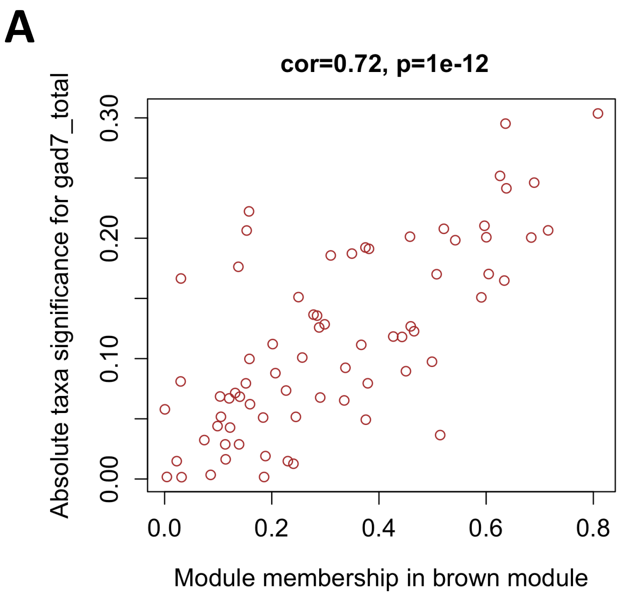

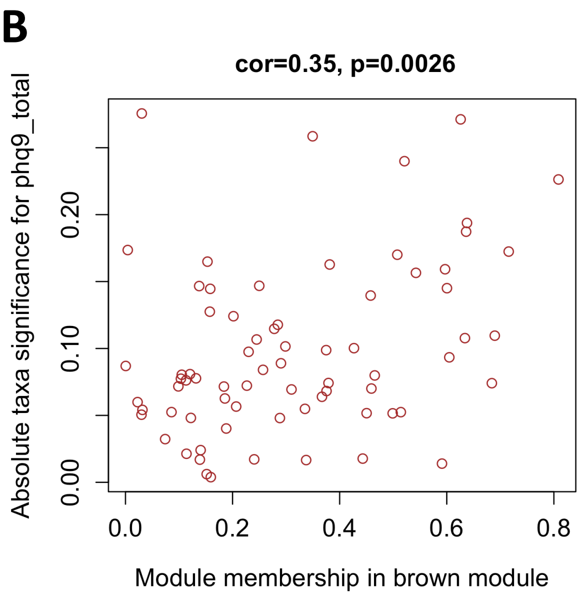

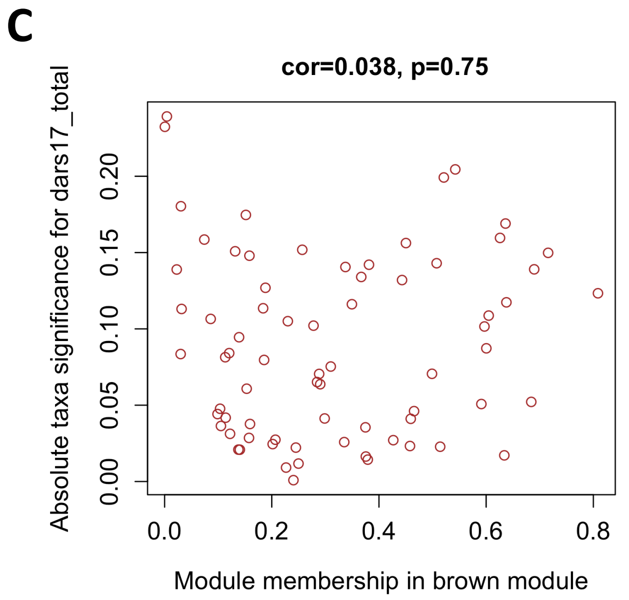

Supplementary Figure S4: Unsigned correlation of absolute taxa significance for clinical traits and covariate-corrected brown module membership. Absolute taxa significance is defined as the unsigned correlation between the taxa and clinical trait. The strength of correlation between absolute taxa significance and module membership has a similar interpretation to taxa significance's correlation with module membership, without considering the direction of microbiome-clinical trait correlation. **A)** GAD7 taxa absolute significance association with module membership. **B)** PHQ9 taxa absolute significance association with module membership. **C)** DARS taxa absolute significance association with module membership.

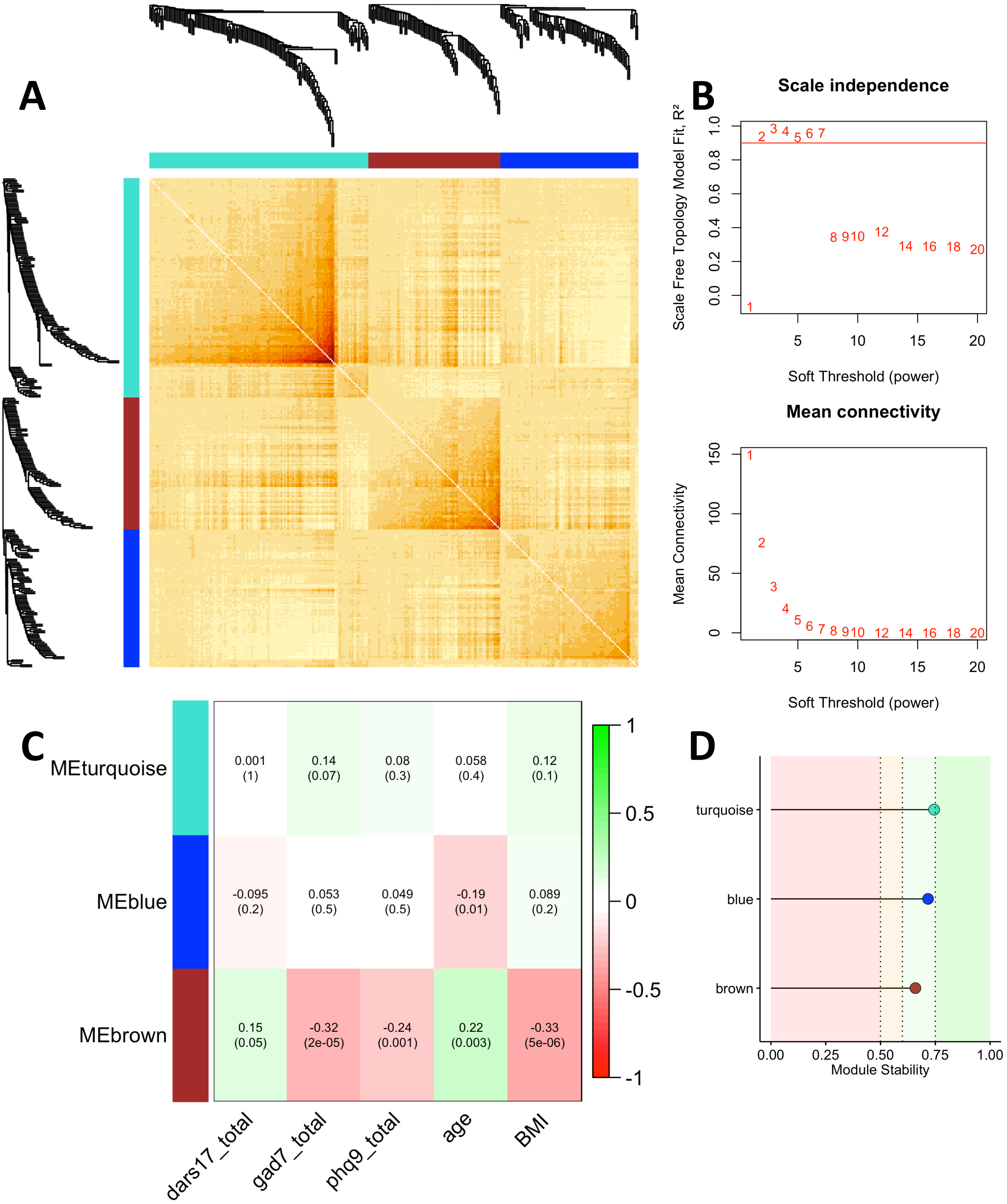

Supplementary Figure S5: Weighted taxon correlation network analysis generated using unadjusted CLR transformed taxa abundance produces similar networks and correlations. **A)** Weighted taxon network visualized using dendrograms and heatmap of Topological Overlap Matrix dissimilarity. Three robust human gut microbial modules are also identified when using unadjusted taxa abundance. **B)** Weighted correlation network soft-threshold parameter selection criteria plots **C)** Correlation of gut microbial networks with clinical mood status. **D)** The uncorrected gut microbial modules are stable. Module stability was calculated using mean Jaccard similarity of one thousand bootstrapped module and original module taxa.

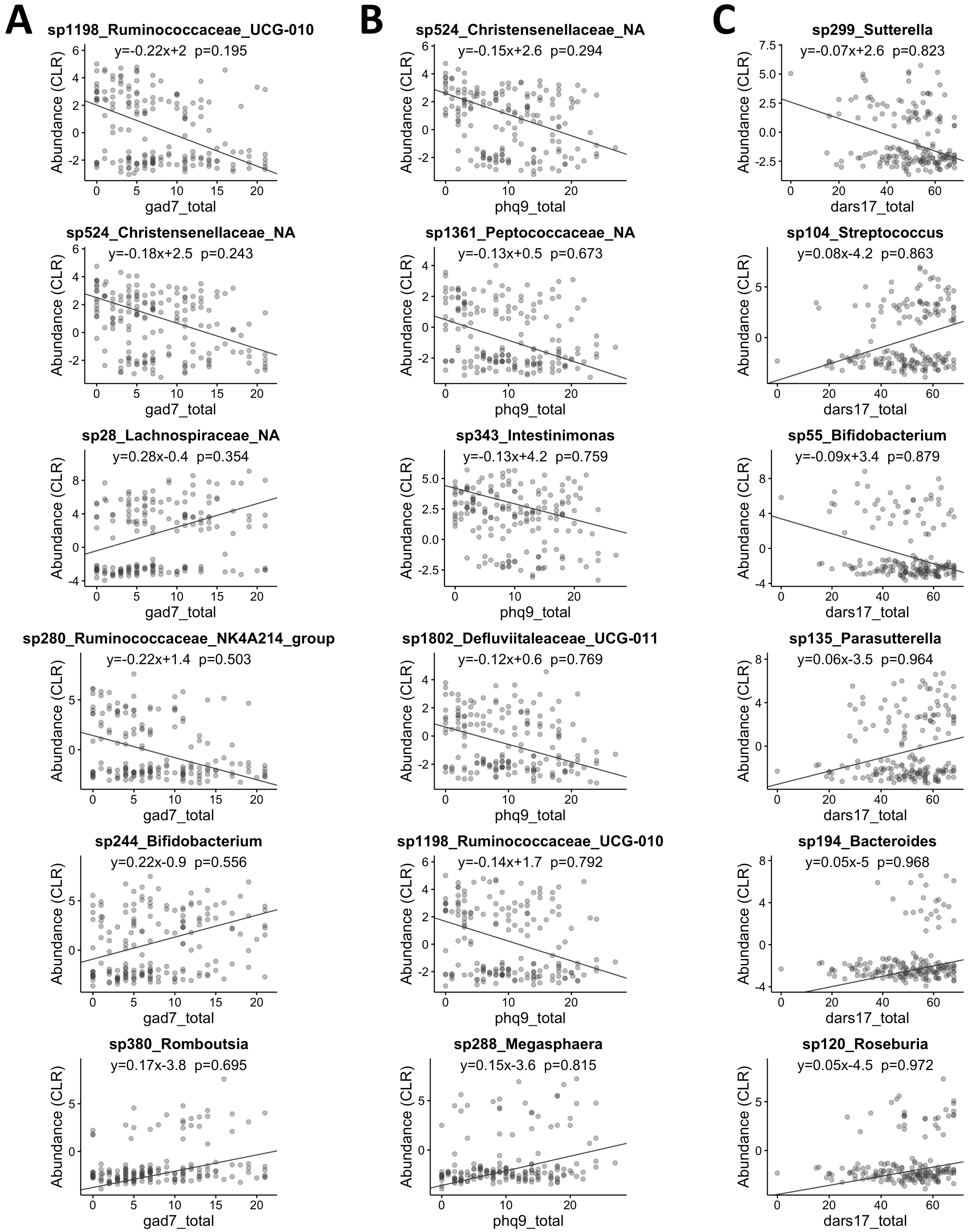

Supplementary Figure S6: Top taxa six associated with age and depression or anxiety clinical scores. Association between clinical trait and taxa abundance was modeled using ALDEx2 linear models. The linear equation and adjusted p-value are reported on each scatter plot. **A)** Top taxa associated with GAD7 clinical depression score. **B)** Top taxa associated with PHQ9 clinical anxiety score. **C)** Top taxa associated with DARS.

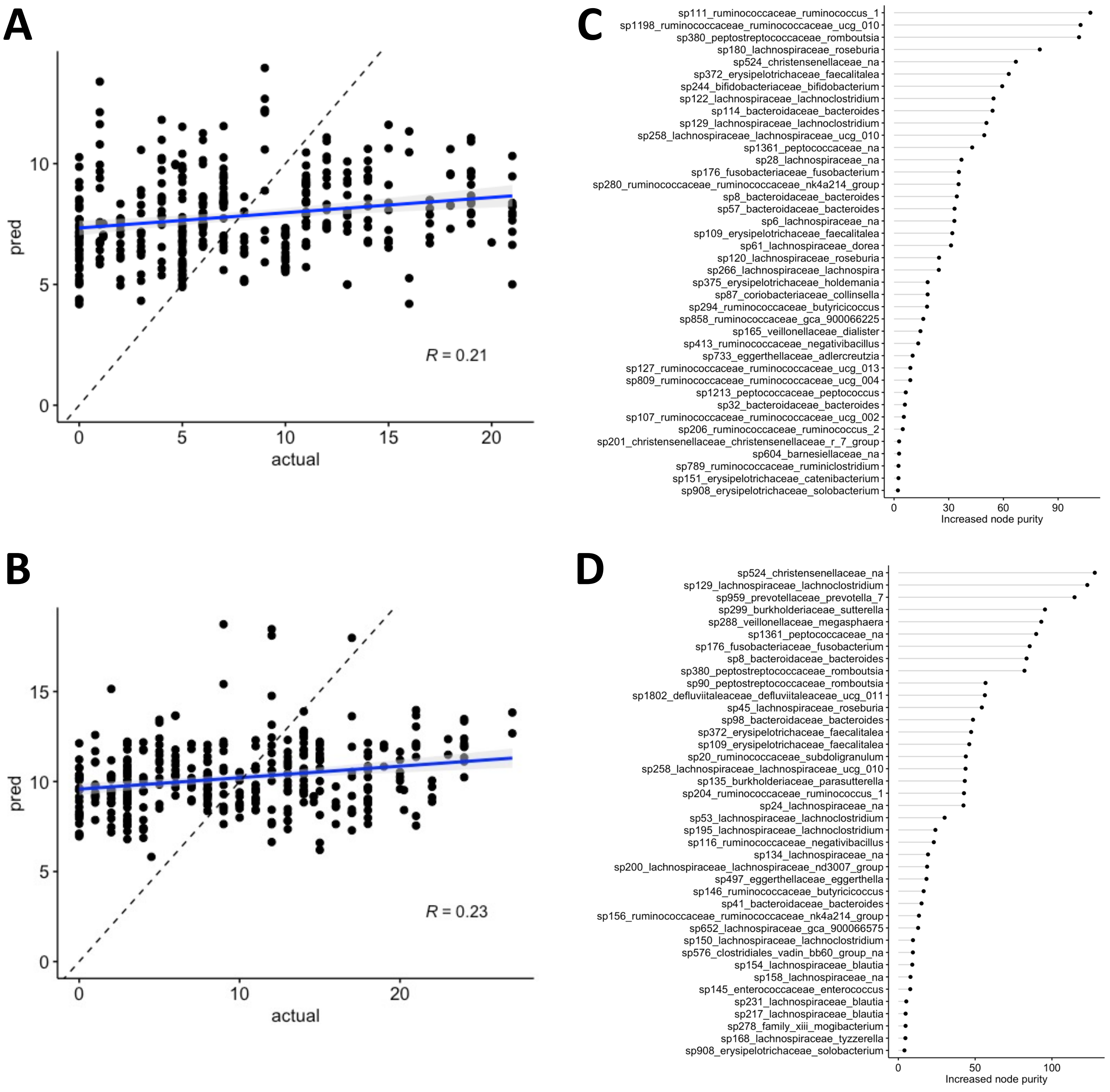

Supplementary Figure S7: Random forest models were trained to predict clinical anxiety and depression scores from gut microbiota composition. Machine learning accuracy was determined by correlating actual and predicted values. The top twenty important predictor variables, bottom 5 important predictor variables, and 15 random predictor variables were plotted in variable importance plots. Several predictor variables that were important for random forest models, were also identified in WCNA and ALDEx2 analyses. **A)** GAD7 total score predictions have poor accuracy using a random forest model trained on human gut microbiome composition. **B)** PHQ9 total score predictions have poor accuracy using a random forest model trained on human gut microbiome composition. **C)** Aggregated predictor variable importance (increased node purity) for GAD7 total score random forest models. **D)** Aggregated predictor variable importance (increased node purity) for PHQ9 total score random forest models.

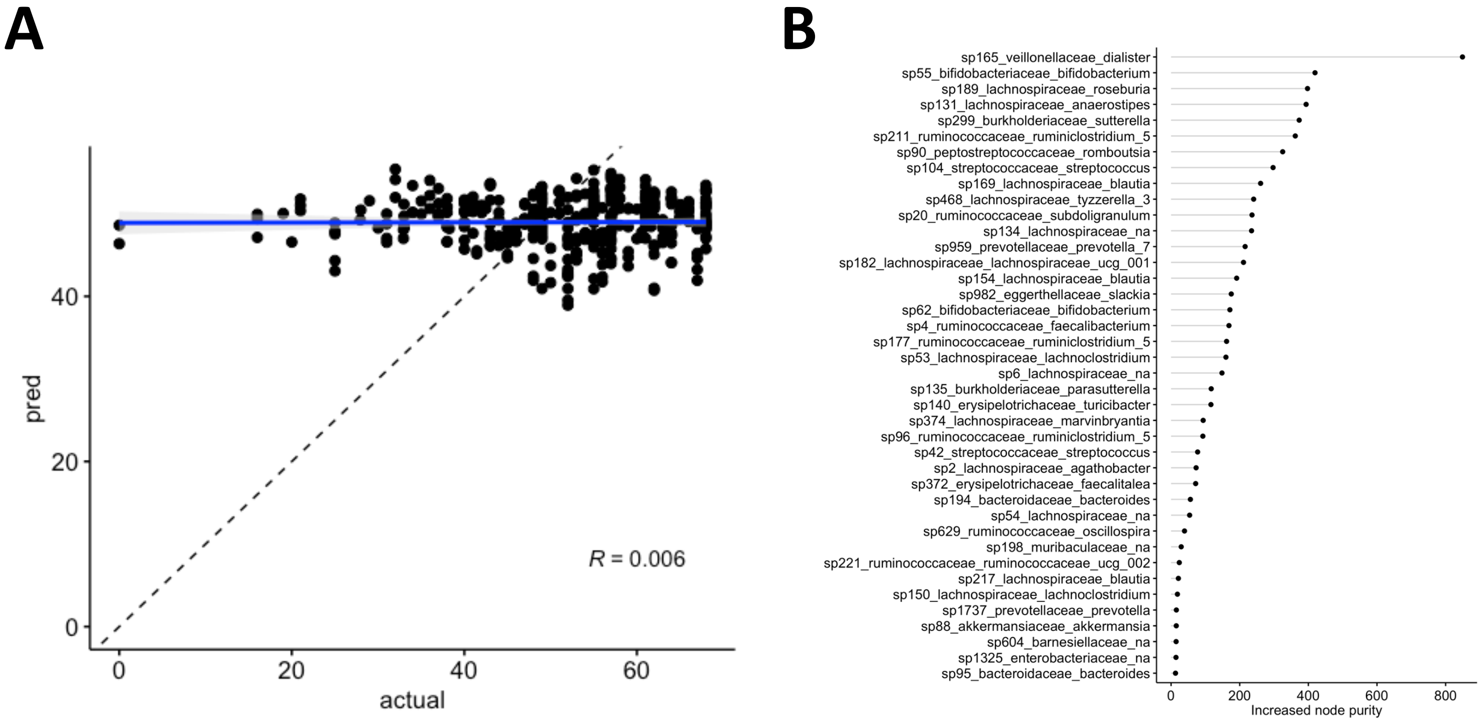

Supplementary Figure S8: Random forest models were trained to predict participant age from gut microbiota composition. Machine learning accuracy was determined by correlating actual and predicted values. The top twenty important predictor variables, bottom 5 important predictor variables, and 15 random predictor variables were plotted in variable importance plots. Several predictor variables that were important for random forest models, were also identified in WCNA and ALDEx2 analyses. **A)** DARS score prediction is completely inaccurate when using a random forest model trained on human gut microbiome composition. **C)** Aggregated predictor variable importance (increased node purity) for random forest models predicting DARS score.
